# Modelling and Analysis of Electrical Potentials Recorded in Microelectrode Arrays (MEAs)

**DOI:** 10.1007/s12021-015-9265-6

**Published:** 2015-03-31

**Authors:** Torbjørn V. Ness, Chaitanya Chintaluri, Jan Potworowski, Szymon Łęski, Helena Głąbska, Daniel K. Wójcik, Gaute T. Einevoll

**Affiliations:** 1 Department of Mathematical Sciences and Technology, Norwegian University of Life Sciences, Ås, Norway; 2 Department of Neurophysiology, Nencki Institute of Experimental Biology of the Polish Academy of Sciences, Warsaw, Poland; 3 Department of Physics, University of Oslo, Oslo, Norway

**Keywords:** Microelectrode array, Modelling, Method of images, Finite element method, Current source density

## Abstract

Microelectrode arrays (MEAs), substrate-integrated planar arrays of up to thousands of closely spaced metal electrode contacts, have long been used to record neuronal activity in *in vitro* brain slices with high spatial and temporal resolution. However, the analysis of the MEA potentials has generally been mainly qualitative. Here we use a biophysical forward-modelling formalism based on the finite element method (FEM) to establish quantitatively accurate links between neural activity in the slice and potentials recorded in the MEA set-up. Then we develop a simpler approach based on the method of images (MoI) from electrostatics, which allows for computation of MEA potentials by simple formulas similar to what is used for homogeneous volume conductors. As we find MoI to give accurate results in most situations of practical interest, including anisotropic slices covered with highly conductive saline and MEA-electrode contacts of sizable physical extensions, a Python software package (ViMEAPy) has been developed to facilitate forward-modelling of MEA potentials generated by biophysically detailed multicompartmental neurons. We apply our scheme to investigate the influence of the MEA set-up on single-neuron spikes as well as on potentials generated by a cortical network comprising more than 3000 model neurons. The generated MEA potentials are substantially affected by both the saline bath covering the brain slice and a (putative) inadvertent saline layer at the interface between the MEA chip and the brain slice. We further explore methods for estimation of current-source density (CSD) from MEA potentials, and find the results to be much less sensitive to the experimental set-up.

## Introduction

Microelectrode arrays (MEAs), that is, substrate-integrated planar arrays of tens to thousands of metal electrode contacts, offer the possibility to record neuronal activity *in vitro* with high spatial and temporal resolution (Taketani and Baudry 2006). MEAs have been successfully used to probe the activity in neuronal cultures (Gal et al. 2010; Tetzlaff et al. 2010; Lambacher et al. 2011; Hierlemann et al. 2011) and retinal (Schneidman et al. 2006; Menzler and Zeck 2011), cerebellar (Frey et al. 2009) and cortical brain slices (Bakker et al. 2009; Miceli et al. 2013). They have also been considered as neuroprosthetic devices (Sekirnjak et al. 2006; Franke et al. 2012).

The high-frequency part of the potentials recorded at the MEA contacts (above some hundred hertz) provides information about the spiking activity of neurons nearby, while the low-frequency part (the local field potential; LFP) also contains information about how the dendrites process synaptic inputs (Pettersen et al. 2012; Buzsáki et al. 2012; Einevoll et al. 2013a). The recorded potentials at the MEA contacts, hereafter referred to as ‘MEA potentials’, are dominated by a weighted sum of contributions from transmembrane currents through the membranes of the neurons (Buzsáki et al. 2012; Einevoll et al. 2013a) in the vicinity of the electrode contacts. The large number of contributing sources makes the interpretation of the MEA recordings complicated. Careful mathematical modelling and analysis are needed to take full advantage of the opportunities that such measurements offer in understanding the signal processing in neurons and neural circuits (Einevoll et al. 2013a; Mahmud et al. 2014). The development of methods for such modelling and analysis becomes even more pertinent with the on-going technological development of MEA chips allowing for recording of potentials at ten thousand or more contact positions (Frey et al. 2009; Lambacher et al. 2011). Such modelling and analysis of MEA signals are the topic of this paper.

Fortunately, the measurement physics of MEA potentials, that is the link between neural activity and what is measured, is in principle well understood: MEA potentials arise from transmembrane currents, and the spread of the signal from each transmebrane current to the various electrode contacts is described by the well-established volume conductor theory (Rall 1962; Rall and Shepherd 1968; Nunez and Srinivasan 2006). The contribution from a point-like transmembrane current source *I* located at ${\mathbf {r}}^{\prime }=(x^{\prime },y^{\prime },z^{\prime })$ to the potential *ϕ* recorded at a point electrode placed at **r**=(*x*,*y*,*z*) in an infinite volume conductor with homogeneous extracellular electrical conductivity *σ*, is given via the simple analytical formula (Rall 1962; Pettersen et al. 2012):

1 $$ \phi(x,y,z) = \frac{I}{4 \pi \sigma \sqrt{(x - x^{\prime})^{2} + (y - y^{\prime})^{2} + (z - z^{\prime})^{2}}}.   $$

Here it is assumed that the conductivity *σ* is *real*, implying that the brain tissue from the extracellular perspective is purely Ohmic, i.e., without capacitive properties. It is further assumed that *σ* is *scalar*, i.e., the same in all directions, and independent of frequency.

The last decade has seen the refinement of a biophysical forward-modelling method based on this volume conductor theory where detailed reconstructed neuronal morphologies have been used in precise calculations of extracellular potentials both spikes (Holt and Koch 1999; Gold et al. 2006, 2007; Pettersen and Einevoll 2008; Pettersen et al. 2008; Schomburg et al. 2012; Thorbergsson et al. 2012; Camuñas-Mesa and Quiroga 2013) and LFPs (Einevoll et al. 2007; Pettersen et al. 2008; Lindén et al. 2010, 2011; Łęski et al. 2013; Lempka and McIntyre 2013; Reimann et al. 2013; Einevoll et al. 2013a, b). The word forward denotes that the extracellular potentials are modeled from neural transmembrane currents; inverse modelling, by contrast, estimates neural activity, e.g., transmembrane currents, from recorded potentials. With this approach, systematic investigations of the link between the recorded potentials and various types of underlying neural activity can be pursued. One obvious application of such modelling is testing data analysis methods, for example for estimation of current-source density (CSD) (Pettersen et al. 2006; Łęski et al. 2007, 2011; Potworowski et al. 2012) or spike-sorting algorithms (Einevoll et al. 2012; Hagen et al. 2015), by use of biophysically detailed model-based ground-truth data.

**Fig. 1:**
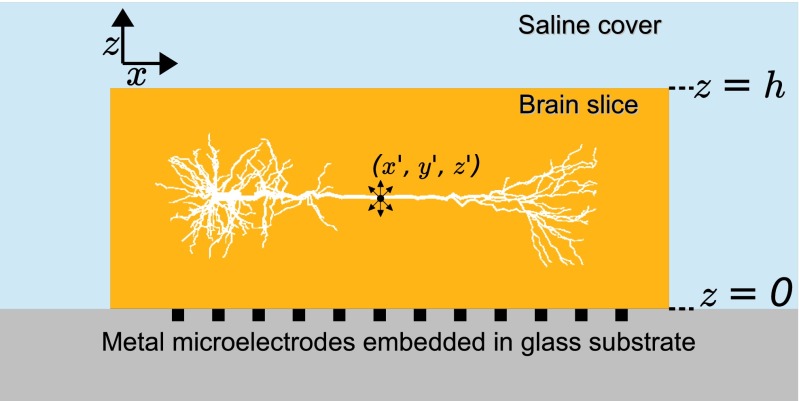
Illustration of MEA electrode, brain tissue slice, and saline bath in experimental set-up. Schematic illustration of present MEA set-up with an *in vitro* slice of brain tissue immersed in saline on top of a substrate-integrated microelectrode array (MEA) (Bakker et al. 2009). The metal electrodes at the MEA chip (embedded in glass substrate) measure the potential set up by the transmembrane currents of the neurons in the brain slice. The dot with protruding arrows represents a point current source at position ($x^{\prime },y^{\prime },z^{\prime }$). Short dotted lines on the right denote the depth coordinates corresponding to the bottom ( *z*=0) and top of the brain slice ( *z*=*h*), respectively

Most such forward-modelling projects have targeted *in vivo* recordings and assumed infinite homogeneous volume conductors, or simple non-homogeneous variations with interfaces between two media with different conductivities (Pettersen et al. 2006). However, the *in vitro* MEA set-up, (Fig. 1), clearly does not correspond to an infinite volume conductor: the MEA chip itself is essentially insulating ( *σ*∼0), yet with a set of small highly conductive metal electrode contacts ($\sigma \sim \infty $) placed on the surface. Further, brain tissue conductivities, *σ*, have been reported in the range 0.2–0.6 S/m (Hämäläinen et al.^1993^; Logothetis et al. 2007; Goto et al. 2010) while the conductivity of the saline bath typically covering brain slices in MEA recordings (Bakker et al. 2009) are about five times larger or more (Grimnes and Martinsen 2008). A further complication is that electrical conductivity in the extracellular medium is not always isotropic. In cortical tissue, for example, it has been found that it is easier for the ions to move in the vertical direction (along the extended apical dendrites) than in the horizontal direction, that is, the vertical extracellular conductivity is about 50 % higher (Goto et al. 2010).

The forward-modelling problem in the general situation with such spatially varying (inhomogeneous) or anisotropic conductivities is, however, always solvable by the computationally more intensive *finite element method (FEM)* (Larson and Bengzon 2013). Here the electrostatic forward problem is solved numerically on a grid, and the link between neural activity and the corresponding recorded potentials can be modelled for any kind of experimental set-up, with explicit modelling of the metal electrode contacts, and any kind of neuronal morphologies, ion membrane channels and synaptic inputs (McIntyre and Grill 2001; Moffitt and McIntyre 2005; Cantrell et al. 2008; Moulin et al. 2008; Joucla and Yvert 2009; Frey et al. 2009; Mechler and Victor 2012; Joucla and Yvert 2012; Lempka and McIntyre 2013; Agudelo-Toro and Neef 2013).

The FEM approach is computationally demanding as one needs to solve for the potential at every grid point in space, not only evaluate it at the electrode contact positions of interest as in Eq. 1. Moreover, for FEM to give accurate results, the spatial mesh has to be very dense around the neuronal transmembrane currents setting up the potential (see Methods section). Alternatively, one can derive a computationally much simpler method based on the *method of images (MoI)* from electrostatics (Jackson 1998). The basic idea behind MoI is to account for the effects of discontinuities of electrical properties at interfaces between dissimilar materials by means of virtual sources. These virtual sources are tailored to satisfy electrical boundary conditions at the material interfaces, thus greatly simplifying the solution of the electrostatic problem. In the present case MoI allows for evaluation of contributions from transmembrane currents to recorded potentials by formulas analogous to Eq. 1 in situations where the current source is embedded in layered volume conductors, for example, for a current source embedded in electrically homogeneous brain tissue sandwiched between an (assumed) fully insulating MEA chip and a thick saline cover (Fig. 1) (Barrera et al. 1978). When applicable, such a scheme is not only much less computationally demanding, it is also much easier to implement than FEM which, for example, requires mesh-making software (see for example (Larson and Bengzon 2013) or fenicsproject.org).

**Fig. 2:**
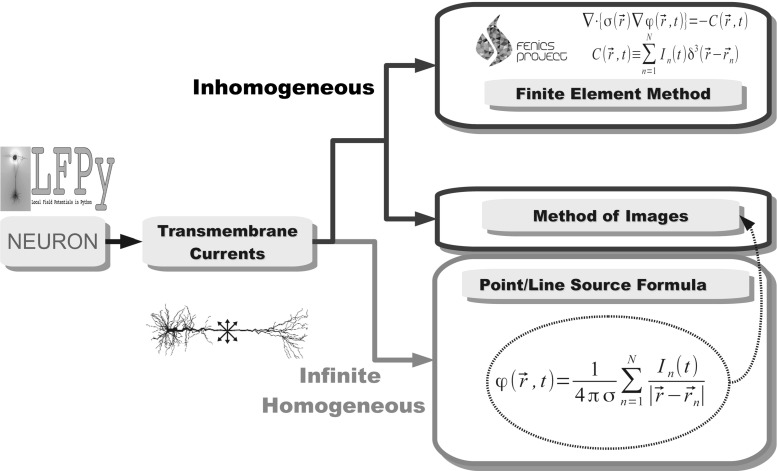
Flowchart of calculation of MEA potentials. Neural activity is simulated using multicompartmental models, here by means of the simulation tool NEURON (Hines and Carnevale 1997), and the transmembrane currents setting up the extracellular potential are extracted using, e.g., the Python tool LFPy (Lindén et al. 2014). If the extracellular medium is assumed to be infinite and homogeneous, the point-source formula in Eq. 1 can be used to compute the potential recorded by an electrode at any position. In the present paper, the analogous line-source formula (Holt and Koch 1999) is also used together with the point-source formula, cf. (Lindén et al. 2014). However, if the extracellular conductivity varies with position around the recording electrodes such as in the MEA set-up (cf. Fig. 1), one has in general to use the more comprehensive *finite element method* (FEM) (Larson and Bengzon 2013). Alternatively, in many situations one may use the *method of images* (Barrera et al. 1978), where simple analytical formulas analogous to Eq. 1 are used to compute the MEA potentials

**Fig. 3:**
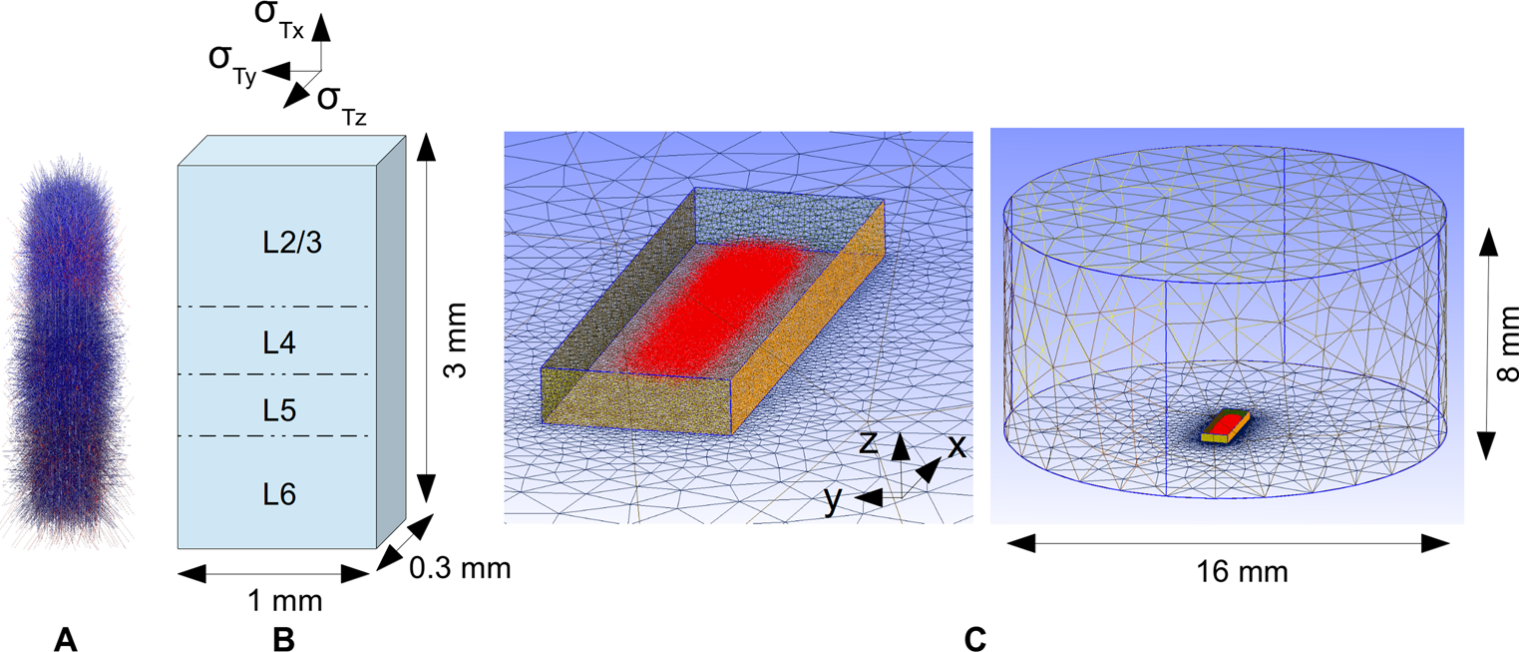
FEM forward-modelling scheme for cortical model network. Illustration of FEM scheme for calculating recorded MEA potentials from activity in a comprehensive model network embedded in an *in vitro* cortical brain slice preparation. **A**: Cells in cortical part of Traub’s model (Traub et al. 2005a), with excitatory cells in blue and inhibitory cells (barely visible) in red; see Table 1 for a description of cell types used. **B**: Sketch of model slice in which network model is embedded, showing spatial dimensions, division into different layers and anisotropic conductivity, i.e., *σ*_*Tx*_ (along apical dendrites of layer 5 pyramidal cells), *σ*_*Ty*_, and *σ*_*Tz*_ (in perpendicular directions). **C**: Collection of red points indicating positions of point current sources due to transmembrane current along the neurons shown in panel **A**; surrounding rectangular box represents the slice. Figure is made with the open source meshing program gmsh (Geuzaine 2009)

In the present paper we develop MoI for the MEA experimental set-up and systematically explore to what extent this approximation can be used to model MEA potentials stemming from neural activity (Fig. 2). Two specific examples of neural activity in model cortical brain slices are considered: the extracellular signature of action potentials (spikes) from a detailed biophysical model based on a morphologically reconstructed neuron (Hay et al. 2011) and LFPs stemming from populations of stylized multicompartmental neurons in a network model of thalamocortical dynamics by Traub et al. (2005a). Next, we test the accuracy of methods for estimating the *current source density* (CSD), i.e., the volume density of transmembrane currents, from recorded MEA potentials (Pettersen et al. 2008; Łęski et al. 2011) taking into account the physical properties of the measurement set-up.

**Table 1:** Cell types used in the thalamocortical model, adapted from (Traub et al. 2005a, b), and the number of cells in every population in the version of the model used here Głąbska et al. (2014). Transmembrane currents from the thalamic cells were not used in the calculation of extracellular potentials

Location	Cell type	Number of cells
layer 2/3	pyramidal regular spiking (RS)	1000
layer 2/3	pyramidal fast rhythmic bursting (FRB)	50
layer 2/3	superficial interneurons — basket,	90 of each
	axoaxonic and low threshold spiking (LTS)	
layer 4	spiny stellate	240
layer 5	pyramidal tufted intrinsic bursting (IB)	800
layer 5	pyramidal tufted regular spiking (RS)	200
layer 5/6	deep interneurons — basket,	100 of each
	axoaxonic and low threshold spiking (LTS)	
layer 6	pyramidal non-tufted RS	500
thalamus	thalamocortical relay (TCR)	100
thalamus	nucleus reticularis (nRT)	100
total		3560

We find that forward modelling with the three-layer MoI approximation (MEA-chip, brain tissue, saline bath) in general accurately reproduces the ground-truth potentials as computed by FEM, also when including the effects of finite-sized electrode contacts. We further find that use of a simpler two-layer MoI approximation (MEA-chip, brain tissue) is sufficient for construction of accurate CSD-estimation methods, in particular, the 2D inverse CSD (iCSD) (Łęski et al. 2011) and kernel CSD (kCSD) (Potworowski et al. 2012) methods.

The computer code for using the new methods, i.e., the new Python toolbox ViMEAPy and kCSD estimation toolboxes in Matlab and Python, is publicly available at the INCF Software repository (software.incf.org).

## Methods

### Electrostatic Forward Modelling

#### Finite Element Method

In the FEM approach both anisotropies and inhomogeneities of the extracellular electrical conductivity in the experimental set-up can be incorporated. Further, microelectrode contacts with physical extensions, i.e., not only point contacts, can be modeled explicitly. Under the quasi-static approximation, the potential *ϕ* in a medium with a position-dependent conductivity tensor $\bar {\sigma }(r)$ is found by solving Poisson’s equation (McIntyre and Grill 2001),

2 $$ \nabla \cdot \bar{\sigma}(\textbf{r}) \nabla \phi(\textbf{r}, t) = -C(\textbf{r}, t),   $$

where *C*(**r**,*t*) is the current source density stemming from the neuronal transmembrane currents.

In the present application, the current sources in the FEM simulations were treated as point sources (Moulin et al. 2008; Moffitt and McIntyre 2005; Joucla and Yvert 2012). The outer boundaries, i.e., glass electrode plate (diameter 16 mm), curved surface of the cylindrical set-up (diameter 16 mm, height 8 mm) containing saline and the saline-air interface (Fig. 3C) were treated as insulating elements (Joucla and Yvert 2009; McIntyre and Grill 2001; Joucla and Yvert 2012), implying homogeneous Neumann boundary conditions,

3 $$ \sigma(\textbf{r}) \nabla \phi(\textbf{r}, t) \cdot \textbf{n} = 0,  $$

where **n** is the unit vector normal to the boundary. This boundary condition is also used for the outer surfaces of the recording electrodes. This corresponds to assuming ‘ideal’ electrodes, i.e., infinite input impedance of the metal electrode contacts. For a justification of these assumptions, see Moulin et al. (2008).

The 3D model geometry consisted of three parts: saline bath, brain tissue, and recording electrodes (Fig. 1). In some simulations a thin layer of saline (10 or 30 m thick) was added between the MEA and the brain slice to test its effect on recorded MEA potentials. The top and outer cylinder boundaries of the saline bath were set to be at ground, i.e., *ϕ*=0, corresponding to Dirichlet boundary conditions (Moulin et al. 2008). The size of the outer cylinder containing the saline bath was increased until further increments did not significantly affect the calculated potentials at the metal electrode contacts. The electrodes were modeled as small volumes penetrating 10 *μ*m into the insulating glass electrode plate, with a very high conductivity, *σ*_*E*_=10^7^ S/m. This conductivity value is in rough agreement with values used by McIntyre and Grill (2001), Moffitt and McIntyre (2005), Cantrell et al. (2008), Mechler and Victor (2012), Joucla and Yvert (2012) and corresponds to platinum electrodes. The results were found to be insensitive to the exact value of this conductivity, however. Note also that the penetration depth of the metal contact (i.e., 10 μm) had no particular significance. It was simply a way to impose metal-like boundary conditions at the electrode contact surface, and a doubling of the depth to 20 *μ*m had no discernible effect on the results. For the brain tissue we used *σ*=0.3 S/m (Hämäläinen et al. 1993; Goto et al. 2010; Nunez and Srinivasan 2006; Logothetis et al. 2007) unless otherwise is stated, while the conductivity of the saline was set to 1.5 S/m (Nunez and Srinivasan 2006; Logothetis et al. 2007).

All FEM simulations were done with the open-source program FEniCS (Logg et al. 2012) software version 1.2.0, with Lagrange P2 elements. The linear systems were solved by the Conjugate Gradient method, and the Incomplete LU factorization preconditioner. The geometry of the MEA set-up was created using the open-source program gmsh (Geuzaine 2009), see Fig. 3 for example set-up.

#### Method of Images

In forward modelling of electrical potentials recorded *in vivo*, the brain is often assumed to be infinite and homogeneous. In this case the extracellular potential at point (*x*,*y*,*z*), generated by transmembrane current *I* located at $(x^{\prime }, y^{\prime }, z^{\prime })$, is given by the point-source formula in Eq. 1. The Method of Images (MoI) (Jackson 1998; Gold et al. 2006; Pettersen et al. 2006) can be used to extend this formula to account for planar steps in the extracellular conductivity at electrode-tissue and tissue-saline interfaces as in the typical experimental MEA set-up (Fig. 1). The basic idea behind MoI is to account for the effects of discontinuities of electrical properties at interfaces between dissimilar materials by means of virtual sources tailored to satisfy electrical boundary conditions at the material interfaces. For the situation with a single point current source positioned in a slab close to an interface with another material with dissimilar electrical conductivity, this can be accomplished by adding a single virtual point source placed at the same distance on the opposite side of the interface. This virtual point source should be placed with the same distance from the interface as the real current source and should be scaled with the factor *W*_12_=(*σ*_1_−*σ*_2_)/(*σ*_1_+*σ*_2_) compared to the real source. Here *σ*_1_ is the conductivity in region 1 containing the real point source, while *σ*_2_ is the conductivity in region 2 on the other side of the interface (Jackson 1998; Gold et al. 2006; Pettersen et al. 2006). Notice, that if we (i) consider the situation with a current source inside a brain slice positioned on top of a MEA electrode and (ii) assume an insulating MEA chip (i.e., *σ*_2_=0), the recorded MEA potential will simply be doubled compared to the analogous situation in an infinite neural-tissue volume conductor.

We are interested in the situation with current sources sandwiched between two planar conductivity-jump interfaces, and in this case the scaling factor *W* is expressed as an infinite series (Barrera et al. 1978; Gold et al. 2006). In the following we denote the conductivity of the saline *σ*_*S*_, the conductivity of the neural tissue *σ*_*T*_, and that of the MEA glass electrode plate *σ*_*G*_. In the general case the potential at position (*x*,*y*,*z*) for a point source at position $(x^{\prime },y^{\prime },z^{\prime })$ positioned inside a slice extending from *z*=0 to *z*=*h* is given by Barrera et al. (1978),

4 $$\begin{array}{@{}rcl@{}} \phi_{PS}(x,y,z) &=& \phi_{h}\left(x-x^{\prime},y-y^{\prime},z-z^{\prime}\right)\\ && +\! \sum\limits_{n=0}^{\infty}\! W_{TG}^{n} W_{TS}^{n} \Big(\! W_{TS}\,\phi_{h}(x\,-\,x^{\prime},\!y\,-\,y^{\prime},z\! +\! z^{\prime}\\ && - 2(n\,+\,1\!)h)+ W_{TG}\, \phi_{h}(x\! -\! x^{\prime},y-y^{\prime},z + z^{\prime} + 2n h)\! \Big)\\ && + \sum\limits_{n=1}^{\infty} W_{TG}^{n} W_{TS}^{n}\big(\phi_{h}(x-x^{\prime},y-y^{\prime},z - z^{\prime} + 2nh)\\ & &+\phi_{h}\left(x\! -\! x^{\prime},y\! -\! y^{\prime},z \! -\! z^{\prime}\! -\! 2nh\right)\!\big), \end{array} $$

with

5 $$\begin{array}{@{}rcl@{}} W_{TG} & \equiv & \frac{\sigma_{T} - \sigma_{G}}{\sigma_{T} + \sigma_{G}}, \end{array} $$

6 $$\begin{array}{@{}rcl@{}} W_{TS} & \equiv &\frac{\sigma_{T} - \sigma_{S}}{\sigma_{T} + \sigma_{S}}, \end{array} $$

and where we have introduced the auxiliary variable *ϕ*_*h*_(*u*,*v*,*w*) corresponding to the potential generated around a current point source *I* positioned at *u*=*v*=*w*=0 in an infinite, homogenous electrical conductor with conductivity *σ*_*T*_:

7 $$ \phi_{h}(u,v,w) \equiv \frac{I}{4 \pi \sigma_{T} \left(u^{2} + v^{2} + w^{2}\right)^{1/2}}.  $$

Here and throughout the rest of the paper, the ground (i.e., *ϕ*=0) of the MoI-computed potentials is assumed to be infinitely far away.

If we further assume the glass electrode plate to have negligible conductivity, i.e., *σ*_*G*_≈0, the expression for the MEA potential recorded at the electrode at *z*=0 from a point current source positioned at $(x^{\prime },y^{\prime },z^{\prime })$, cf. (Fig. 1), simplifies to,

8 $$\begin{array}{@{}rcl@{}} \phi_{PS}(x,y,0) & = & 2 \, \phi_{h}\left(x-x^{\prime},y-y^{\prime},-z^{\prime}\right)\\ & &+ 2 \sum\limits_{n=1}^{\infty} W_{TS}^{n} \big( \phi_{h}(x\,-\,x^{\prime},y\! -\!y^{\prime},\!-z^{\prime} + 2nh)\\ && + \phi_{h}(x-x^{\prime},y-y^{\prime},-z^{\prime} - 2nh) \big).\\ \end{array} $$

Equation 8 gives the MoI expression for the recorded MEA potential assuming the transmembrane currents to be point sources. For the commonly used alternative line-source approximation (Holt and Koch 1999; Gold et al. 2006; Pettersen and Einevoll 2008; Lindén et al. 2014), where the transmembrane currents are assumed evenly distributed along the axes of cylindrical compartments with an axis stretching from $(x_{0}^{\prime }, y_{0}^{\prime }, z_{0}^{\prime })$ to $(x_{1}^{\prime }, y_{1}^{\prime }, z_{1}^{\prime })$, we obtain

9 $$\begin{array}{@{}rcl@{}} \phi_{LS}(x,y,0) & = &2 \, \tilde{\phi}_{h}\left(x-x_{0}^{\prime},y-y_{0}^{\prime},-z_{0}^{\prime}\right)\\ & &+ 2 \sum\limits_{n=1}^{\infty}W_{TS}^{n} \left(\tilde{\phi}_{h}\left(x-x_{0}^{\prime},y\! -\!y_{0}^{\prime},- z_{0}^{\prime}+2nh \right)\right.\\ &&+\left. \tilde{\phi}_{h}\left(x-x_{0}^{\prime},y-y_{0}^{\prime},-z_{0}^{\prime}-2nh \right)\right), \\ \end{array} $$

where the auxiliary potential variable $\tilde {\phi }$ is introduced as

10 $$ \tilde{\phi}_{h} (u,v,w) \equiv \frac{I}{4 \pi \sigma_{T} \varDelta s} \ln\left( \frac{\varDelta s^{2} - \gamma(u,v,w) + \varDelta s \sqrt{\varDelta s^{2} - 2\gamma(u,v,w) + \rho(u,v,w)^{2}}} {- \gamma(u,v,w) + \rho(u,v,w) \varDelta s}\right),  $$

where

11 $$ \gamma(u,v,w) \equiv u\left(x_{1}^{\prime}-x_{0}^{\prime}\right) + v\left(y_{1}^{\prime}-y_{0}^{\prime}\right) + w\left(z_{1}^{\prime}-z_{0}^{\prime}\right),  $$

12 $$ \rho(u,v,w) \equiv \left( u^{2} + v^{2} + w^{2} \right)^{1/2}\;,  $$

and

13 $$ \varDelta s \equiv \left( \left(x_{1}^{\prime}-x_{0}^{\prime}\right)^{2}+\left(y_{1}^{\prime}-y_{0}^{\prime}\right)^{2}+\left(z_{1}^{\prime}-z_{0}^{\prime}\right)^{2} \right)^{1/2}  $$

is the length of the axis of the cylindrical compartment.

The MoI formulas for *ϕ*(*x*,*y*,0) in Eqs. 8 and 9 involve a sum over an infinite series of terms. In practice, however, the series converges fast, and the number of required terms turned out to be computationally unproblematic. Unless otherwise indicated, we summed over 20 terms in this study.

We have not found an analytic solution for the three-layered set-up with arbitrary anisotropies in the extracellular conductivities in the three slabs (Eskola 1988; Wait 1990; Li and Uren 1997; Mele 2001). However, the MoI approach can still be used for interfaces between layers where (i) one layer has zero conductivity (MEA electrode plate in our case) and (ii) the relative planar anisotropy, i.e., *σ*_*x*_/*σ*_*y*_, is the same in the other two layers (Wait 1990). To allow for the use of analytical MoI formulas in the present situation, we thus made the *ad hoc* approximation of assuming the saline conductivity in the planar directions to have the same relative anisotropy as the cortical tissue, i.e., *σ*_*Sx*_/*σ*_*Sy*_=*σ*_*Tx*_/*σ*_*Ty*_=*α*_*a*_, where *α*_*a*_ is a scalar quantifying the anisotropy of the tissue. (The modest error typically resulting from this *a priori* unphysical assumption is discussed in detail in Results.) In cortical brain tissue the conductivity is typically larger in the direction of the extended apical dendrites (Fig. 1), here defined as the *x*-direction, than in the two lateral directions (here *y* and *z*). Goto et al. (2010) estimated differences between conductivity in the two directions of up to 50 %, cf. Table 2. Thus, in the MEA set-up, there will typically be higher conductivity in one of the planar directions than in the other. We further used the same saline conductivity in the *y*- and *z*-directions, i.e., *σ*_*Sz*_=*σ*_*Sy*_.

**Table 2:** Vertical positions of the cortical layers in the model accompanied by the layer-specific electrical conductivities as reported by Goto et al. (2010). *σ*_*Tx*_ is the conductivity parallel to the apical dendrites of the layer 5 pyramidal cells, while *σ*_*Ty*,*Tz*_ is the conductivity in the direction perpendicular to them. Mean values obtained from five rats are listed as well as their standard deviations

Layer	Layer depth ( *μ*m)	*σ*_*Tx*_ (S/m)	*σ*_*Ty*,*Tz*_ (S/m)
2/3	0 to −400	0.319 ± 0.043	0.231 ± 0.056
4	−400 to −700	0.325 ± 0.067	0.240 ± 0.093
5	−700 to −1200	0.353 ± 0.063	0.228 ± 0.047
6	−1200 to −1700	0.294 ± 0.062	0.268 ± 0.067

This gives the following point-source MoI formula for the anisotropic case:

14 $$\begin{array}{@{}rcl@{}} \phi_{PS,a}(x,y,0) & = & 2 \, \phi_{h,a}\left(x-x^{\prime},y-y^{\prime},-z^{\prime}\right)\\ &&+ 2 \sum\limits_{n=1}^{\infty} W_{TS,a}^{n}\left(\phi_{h,a}\left(x\! -\! x^{\prime},y\! -y^{\prime},-z^{\prime} +2nh\right)\right.\\ &&+\left.\phi_{h,a}\left(x-x^{\prime},y-y^{\prime},-z^{\prime} -2nh\right)\right),\\ \end{array} $$

where (Wait 1990)

15 $$ W_{TS,a} \equiv \frac{\sqrt{\sigma_{Tx}\sigma_{Ty}} - \sqrt{\sigma_{Sx}\sigma_{Sy}}} {\sqrt{\sigma_{Tx}\sigma_{Ty}} + \sqrt{\sigma_{Sx}\sigma_{Sy}}}\;,   $$

and a new auxiliary potential *ϕ*_*h*,*a*_, stemming from the anisotropic point-source equation (Nicholson and Freeman 1975; Eskola 1988; Wait 1990), has been introduced:

16 $$ \phi_{h,a}(u,v,w) \equiv \frac{I}{4 \pi \left( \sigma_{Ty} \sigma_{Tz}u^{2} + \sigma_{Tx} \sigma_{Tz}v^{2} + \sigma_{Tx} \sigma_{Ty} w^{2}\right)^{1/2}}.  $$

Equations 15 and 16 can be further simplified by use of *σ*_*Ty*_=*σ*_*Tz*_=*σ*_*Tx*_/*α*_*a*_ and *σ*_*Sy*_=*σ*_*Sz*_=*σ*_*Sx*_/*α*_*a*_, yielding

17 $$ W_{TS,a} = \frac{\sigma_{Ty} - \sigma_{Sy}} {\sigma_{Ty} + \sigma_{Sy}}\;.  $$

and

18 $$ \phi_{h,a}(u,v,w) = \frac{I}{4 \pi \sigma_{Ty} \left(u^{2} + \alpha_{a} v^{2} + \alpha_{a} w^{2}\right)^{1/2}}.  $$

The finite size of the electrode contacts were incorporated into the MoI framework by averaging computed potential values at 100 randomly chosen points with uniform planar density across the hypothetical electrode surface (Lindén et al. 2014). It was verified that increasing the number of averaging points beyond 100 did not significantly affect the present results.

### Applications to Experimental Situations

#### MEA Spike from Single Neuron

To investigate the MEA measurement of a spike, i.e., the extracellular signature of a neuronal action potential, in the model slice, we used a multicompartmental model of a layer-5 pyramidal neuron from Hay et al. (2011), downloaded from ModelDB (senselab.med.yale.edu/modeldb/, model accession number 139653). Spiking was induced in the model by increasing the reversal potential for the passive leak current of the entire cell from -90 mV to -55 mV prior to onset of the simulation, so that the model spontaneously generated a spike.

The neuronal simulations were done with the simulation tool NEURON (www.neuron.yale.edu), facilitated by the Python package LFPy (Lindén et al. 2014). The transmembrane currents and positions of the compartments were subsequently used to calculate the extracellular potential by means of MoI using the line-source approximation with and without the saline bath included in the simulations.

#### MEA Potential from Cortical Neural Network

To generate extracellular potentials stemming from neural populations, we considered the thalamocortical model of Traub et al. (2005a, b), to our knowledge the most comprehensive publicly available model of a thalamocortical network based on multicompartmental neuron models. The model contains 3560 stylized multicompartmental cells divided into 14 populations as described in Table 1. To calculate the extracellular potentials in the cortex, we excluded contributions from transmembrane currents from the thalamic cells. This left a total of 3360 cells with 211490 compartments contributing to the simulated recordings of MEA potentials.

The original version of the model was developed in IBM FORTRAN (ModelDB accession number 45539) (Traub et al. 2005a, b). The version we used was derived by combining a NEURON implementation (ModelDB accession number 82894) with the full 3D cell morphologies exported from the NeuroML version (ModelDB accession number 127353) (Gleeson et al. 2007). Further, axonal gap junctions were removed (default in the NEURON version). To facilitate the computation of extracellular potentials we added the possibility of recording currents from all the compartments and distributed the cortical cells within a slab mimicking a cortical brain slice preparation (Głąbska et al. 2014). A version of the code used for an example simulation and generation of current sources used here for computation of LFP is available at github.com/hglabska/Thalamocortical_imem.

The original Traub model does not specify the spatial positions of the neurons which are needed to calculate extracellular potentials. We therefore placed the cells so that the somas of every population were distributed randomly with uniform distribution in cylindrical boxes of diameter 400 μm and heights corresponding to the vertical extent of the layers as described in Table 2, see also Fig. 3A. The cylindrical axis was in all cases oriented in parallel to the MEA surface, but three different elevations within the tissue slab in the MEA set-up were considered: (i) cylinder axis in the middle of the slice, (ii) axis shifted 25 *μ*m towards the MEA surface, or (iii) shifted 25 *μ*m towards the saline bath. To fit inside the model slice, the spatial extension of the dendrites of the neurons in the population was reduced by a factor of four in the vertical (*z*) direction, resulting in the elongated distribution of point sources that is shown in Fig. 3. As the main purpose of these network simulations was to investigate the effects of the MEA set-up on the recorded LFP (as well as the estimation of current-source density (CSD), see below), we do not think that this focusing of the dendritic transmembrane currents in the *z*-direction is of significance for the overall conclusions. Note also that this spatial compression of the dendrites was applied after the network simulation, and thus only affected the predicted MEA potentials, not the network dynamics.

To calculate the MEA potential we simulated 600 ms of the network activity in response to injection of oscillatory current ( *f*=50 Hz frequency, *I*_inj_=2 nA amplitude) into thalamocortical relay cells starting at 100 ms from the onset of the simulation:

19 $$ I(t) = I_{\text{inj}} \sin (2 \pi f (t-t_{0})) \varTheta(t-t_{0}),  $$

where *Θ*(*t*) is the Heaviside function, and *t*_0_=100 ms. We stored the transmembrane currents of all the compartments in all the neurons. The results for the first 100 ms of simulation were discarded to avoid artifacts from the initial conditions. We used the point-source approximation, i.e., the calculated transmembrane currents were assumed to be point sources positioned at the midpoints of the axes of the cylindrical compartments. Figures 13 and 14 show results for one representative snapshot in time ( *t*=221 ms, i.e., 121 ms after the onset of the stimulation).

**Fig. 4:**
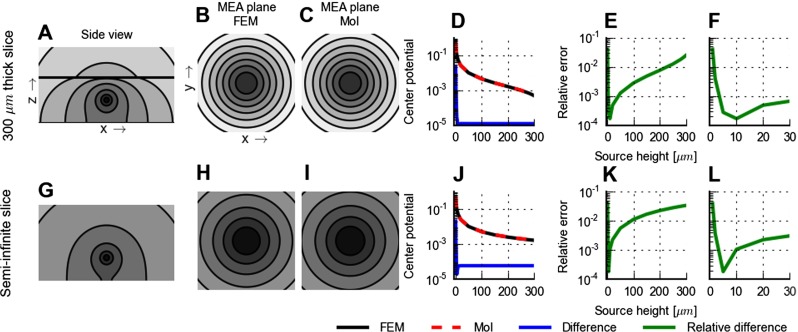
Illustration and comparison of modelling schemes for electrical potentials. **A-C**: Electrical potential from a current point source positioned in the middle of a *h*=300 *μ*m thick brain slice sandwiched between a fully insulating MEA plane and an (infinitely thick) saline cover. The slice is electrically isotropic and homogeneous and covers the entire ( *x*,*y*) plane, and *σ*_*S*_/*σ*_*T*_=1.5/0.3=5. Panel **A** shows a contour plot of the FEM potential in a 900 × 450 *μ*m section of ( *x*,*z*)-plane extending from *z*=0 (MEA plane); shown plane is displaced 10 *μ*m from the current source in the *y*-direction. The interface between slice and saline is indicated by black horizontal line. The contour plot is logarithmic, and the potential is reduced by 50 % between contour lines. Panels **B** and **C** show contour plots of the potential in a 1000 ×1000 *μ*m section of MEA-plane (( *x*,*y*)-plane for *z*=0) with the ( *x*,*y*)-position of the current source in the center of the square section; **B**: finite-element method, FEM; **C**: method of images, MoI, for point electrode, Eq. 8. The contour plots are logarithmic with the potential reduced by 25 % between contour lines. Panel **D** shows FEM and MoI predictions of the center MEA potential, i.e., potential directly below the point current source in the MEA plane, as a function of source distance. (This would correspond to the lead-field of an ideal MEA point electrode positioned below the current source, i.e., with the same *x* and *y* coordinates.) Potentials are normalized to the value found for a source height of 1 *μ*m, the smallest distance considered, which also corresponds to the mesh size in the FEM simulations. Blue curve shows the magnitude of the difference between the FEM and MoI predictions. Panel **E** shows the corresponding relative difference, i.e., the magnitude of the difference between the FEM and MoI predictions divided by the MoI prediction, while panel **F** shows a subset of the results in **E** highlighting the relative difference for current sources close to the MEA plane. **G-L**: Same as panels **A-F** but with a semi-infinite slice, i.e., $h \rightarrow \infty $. FEM potentials are set to zero at simulation grid boundaries; MoI potentials are set to zero at infinity

**Fig. 5:**
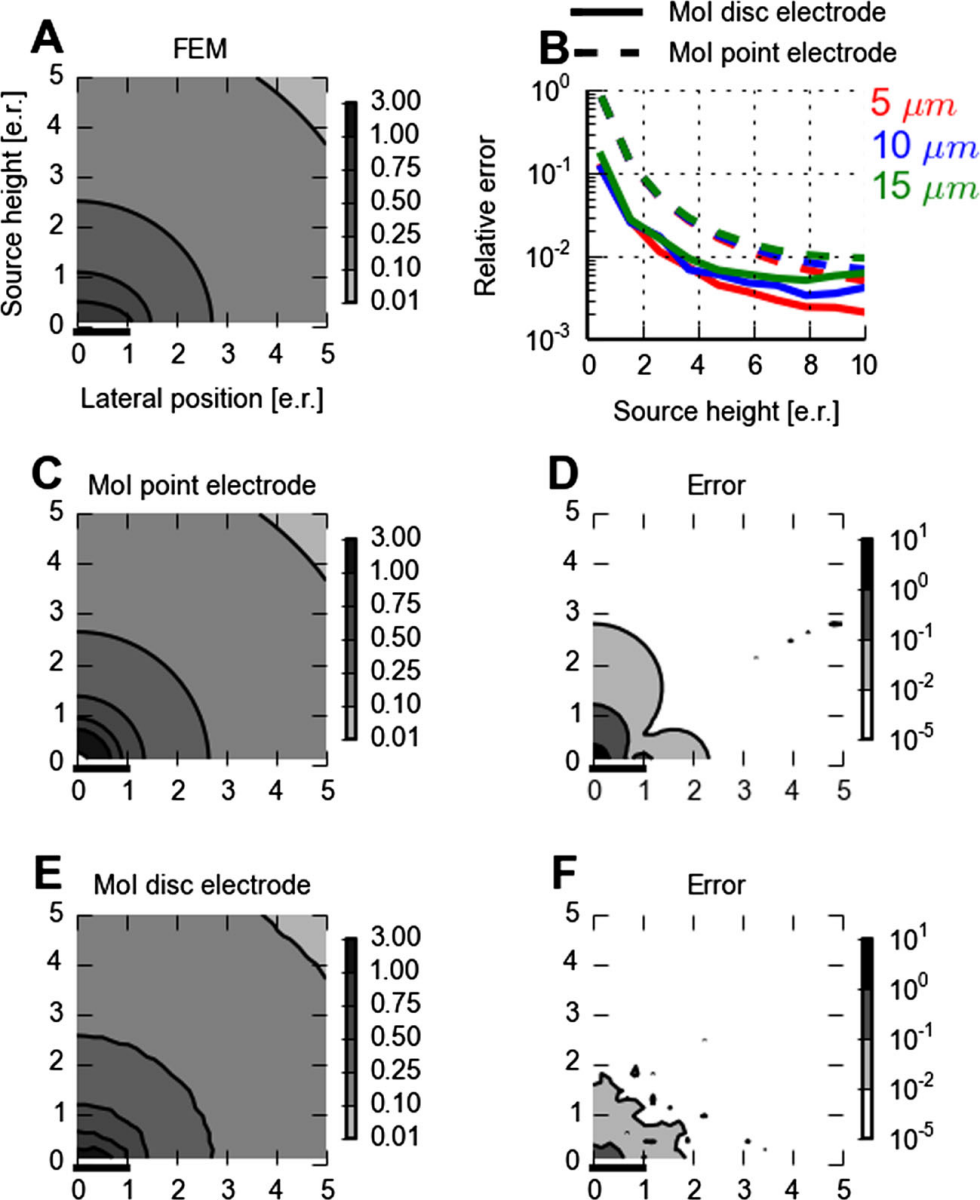
Effects of electrode size on MEA lead field. Panel **A** shows the lead field for a 300 *μ*m brain slice covered with saline for a MEA disc electrode. Potentials are computed by means of FEM. The flat, circular electrode has a radius of 10 *μ*m and is placed in the (*x*,*y*)-plane (i.e., *z*=0) with its center at *x*=*y*=0 as indicated by the thick horizontal line at the origin denoting half of the lateral electrode extension. The contour plot shows the lead field as a function of source height (*z*) and lateral positions (*x*) at *y*=0 in units of electrode radii. Results are normalized to the largest computed value (found for *x*=*y*=*z*=0). Panels **C** and **E** show the lead fields for the point-electrode (8) and disc-electrode MoI approximations (22), respectively. The results are normalized to the maximum value for the lead field obtained with FEM (panel **A**) and can thus be larger than unity, as they are for the point-electrode MoI results for sources close to the recording electrode. The corresponding deviations, i.e., absolute value of differences, between MoI and FEM results are shown in panels **D** and **F**, respectively. In panel **B** the relative deviations in the lead field, i.e., deviations divided by the FEM result, are shown along the *z*-axis (i.e., *x*=*y*=0) for three different disc-electrode radii: 5, 10 and 15 *μ*m. In all panels lengths are measured in units of electrode contact radii (e.r.)

### Analysis Methods — kCSD

We modified the original kCSD method (Potworowski et al. 2012) by replacing the point-source model for an infinite homogeneous volume conductor in Eq. 1 with the MoI point-source forward model, Eq. 8. As in Potworowski et al. (2012) we assumed the sources to be distributed homogeneously throughout the slice thickness, i.e., in the *z*-direction. This allows for analytical integration of the forward model in the *z*-direction. As a result, we obtain the following formula for the bases in the space of MEA potentials (analogous to Eq. (2.22) in Potworowski et al. (2012)):

20 $$\begin{array}{@{}rcl@{}} b_{i}(x,y) & = & \frac{1}{2\pi\sigma_{T}} {\int}_{-\infty}^{\infty}\mathrm{d}x^{\prime}{\int}_{-\infty}^{\infty} \mathrm{d}y^{\prime} \left[ \text{arsinh} \frac{h}{L}\right.\\ & &+ \sum\limits_{n=1}^{\infty} W_{TS}^{n} \left.\left( \text{arsinh}\frac{h-2hn}{L}\right.\right.\\ &&+\left.\left. \text{arsinh}\frac{h+2hn}{L} \right) \right] \widetilde{b}_{i}(x^{\prime}, y^{\prime}), \end{array} $$

where $L = \sqrt {(x-x^{\prime })^{2} + (y-y^{\prime })^{2}}$.

In this work we used two variants of kCSD. In the first variant, denoted kCSD _0_, we omit the series in Eq. 20, physically corresponding to assuming an insulating MEA electrode plane and a semi-infinite slice (no saline bath). In the second variant, denoted kCSD _20_, we kept the first 20 terms in the sum in Eq. 20, physically corresponding to including the MEA electrode plane, the brain slice, and the saline bath in the forward model.

### Data Analysis

For quantitative comparison of results we (at times) used the root-mean-square (RMS) signal, i.e.,

21 $$ A_{RMS} = \sqrt{\sum\limits_{i=1}^{N} \frac{{A_{i}^{2}}}{N}}  $$

where the sum goes over all *N* computed signals *A*_*i*_ in a grid of electrodes in the MEA plane. The signal *A*_*i*_ is either (i) the predicted MEA potential itself or (ii) the difference between MEA potentials computed by means of two different methods.

### Software

A simple and efficient MoI solver for calculation of extracellular potentials in the *in vitro* brain slice setting was implemented in Python, with the additional use of Numpy and Cython for computational efficiency. It is made freely available under the name ViMEAPy (*Virtual MEA signals in Python*) at software.incf.org. A version of 2D kCSD scripts including MoI corrections is available upon request from the authors and will be included in the next official release available at software.incf.org/software/kcsd. Also the scripts for FEM modelling of the potential propagation in MEA set-up are available upon request from the authors. All software is released under the GNU General Public License.

## Results

The results come in four parts: We first focus on the verification of the application of the method of images (MoI) in the context of forward modelling of MEA potentials by comparing with results from use of the finite element method (FEM). This is done both for idealized point-electrode contacts and for disc-electrodes with finite electrode radii. Then, we explore the effects on the recorded MEA potentials from (i) anisotropic and inhomogeneous electrical conductivities within the brain slice and (ii) the surrounding high-conductivity saline bath. Next, we show results for two specific neural applications, i.e., how the experimental set-up affects the MEA potentials recorded from (i) a single spiking pyramidal neuron and (ii) network activity in a population of neurons, respectively, embedded in a cortical brain slice. Finally, we investigate the inverse problem, i.e., how to best estimate the current-source density (CSD) in a cortical brain slice from recorded MEA potentials.

### Verification of MoI Scheme

To illustrate the spatial distribution of potentials set up by a neural current source in a MEA setting, we show in Fig. 4A–B contour plots of the potentials around a current source placed in the middle of a brain slice of thickness 300 *μ*m covered by saline. The side view in panel A shows that while the potential decays Coulomb-like (i.e., inversely with distance) close to the point source, both the insulating MEA layer at the bottom and the saline cover at the top distort the potentials close to the interfaces. For our purposes the potential recorded in the MEA plane is most important, and panel B shows the circularly symmetric distribution of potentials in this plane. For comparison we show in panels G and H the corresponding potentials in the case of a semi-infinite slice, i.e., without any saline cover.

#### MoI vs. FEM

In this paper the FEM scheme is generally used to generate ground-truth data against which approximate MoI results can be compared. However, for the examples considered in Fig. 4, exact analytical solutions for the potentials can be obtained by means of MoI: for potentials recorded with ideal point electrodes in the MEA plane, the forward-modelling formula is given by Eq. 8. While FEM in principle can solve the electrostatic forward problem for arbitrarily complicated spatial geometries and distributions of electrical conductivities, the accuracy of the solution will depend on the chosen underlying mesh. We thus took advantage of the available analytical solutions to test the accuracy of the present FEM implementation itself. The prediction of the MEA-plane potentials from the MoI formula in the situation with the 300 *μ*m slice, is shown in Fig. 4C. A visual comparison reveals essentially no difference with the corresponding FEM results depicted in panel B.

In modelling of EEG signals the concept of *lead field* refers to the forward-model link between neural dipoles and the potential recorded at EEG electrodes and in particular the dependency of this link on the dipole position (Malmivuo and Plonsey 1995). Here we correspondingly compute a *MEA lead field* describing the electrical potential that will be measured at a particular MEA electrode contact by point current sources placed at different positions. In panels D–F of Fig. 4 we compare MoI and FEM results for this lead field, i.e., the (point-electrode) MEA potential from a single current source positioned at different heights above the recording contact. Also here a close agreement between FEM and MoI results is seen: The largest relative errors are seen in panel E to occur for sources placed close to the MEA recording plane. For sources placed further out, i.e., between than 5 and 30 *μ*m above the MEA plane, the relative error is seen in panels E and F to be very small (less than 0.1 %).

The relative error is seen in panels E and F to be at a minimum for a source height of about 10 *μ*m, and then increase again with larger source heights. This effect stems from somewhat different implementations of the grounding, i.e., the enforcement of a zero electrical potential, in FEM and MoI. The FEM simulation is by its nature spatially confined to the overall size of the simulation grid, and in the present simulations the potential is set to zero at the grid boundaries. For the MoI calculations we always used zero at infinity as a boundary condition. This difference in boundary conditions gives a constant difference between the MoI and FEM results of about 10^−5^ for source heights larger than about 10 *μ*m, panels D–F. Since the lead field itself is decreasing with increasing source height, the relative difference will thus increase for heights beyond 10 *μ*m. However, the relative error never gets larger than a few percent.

For the case with the semi-infinite slice the exact analytical MoI solution in Eq. 8 simplifies even further: with *W*_*TS*_=0, the MEA potential is simply twice the Coulomb-like potential around a point current source in an infinite slice, i.e., in an infinite volume conductor. An excellent agreement between FEM and the exact MoI lead-field results is seen also here, cf. Fig. 4G-L.

We thus conclude that in the present situation the FEM simulations are generally very accurate as long as the distances considered between the current sources and recording potential are, say, a factor five or more larger than the minimum mesh size.

#### Electrodes with Physical Extension

So far we have only considered ideal point electrodes, i.e., hypothetical electrodes that have no physical extension and thus do not disturb the electrical potential in the vicinity of the electrode contact. However, real MEA electrodes, hereafter referred to as *disc electrodes*, of course have a physical extension, typically with diameters in the range between 5 and 30 *μ*m (Bakker et al. 2009; Frey et al. 2009; Lambacher et al. 2011; Heim et al. 2012). Close to the electrode surface the electric potential will be affected by the high-conductivity electrode contact (McIntyre and Grill 2001; Moffitt and McIntyre 2005; Moulin et al. 2008). Consequently, for current sources positioned close to the surface, the recorded MEA potential will be affected by the size and shape of the electrode contact. Such disc electrodes appear to measure the average potential across the uninsulated electrode surface (Moulin et al. 2008; Nelson and Pouget 2010). A natural approximation for modelling the potential recorded by disc electrodes is thus to extend the point-electrode MoI approximations in Eqs. 8, 9, or 16 by computing the average potential *Φ* across the disc-electrode surface *S* (Moulin et al. 2008; Lindén et al. 2014), i.e.,

22 $$ \varPhi = \frac{1}{A_{S}} \iint_{S} \phi(\mathbf{u},0) \,d^{2} u \approx \frac{1}{m} \sum\limits_{i=1}^{m} \phi(\textbf{u}_{i},0).  $$

Here **u** is a two-dimensional vector describing positions of the tissue-facing surface *S* of the electrode contact, and *A*_*S*_ is the surface area. In the second step, the surface integral is approximated as a sum over *m* positions chosen randomly with a uniform planar distribution, covering the electrode surface, corresponding to numerical Monte Carlo integration.

In Fig. 5 we compare the accuracy of using the point-electrode (panel C, Eq. 8) and disc-electrode MoI approximations (panel E, Eq. 22) against ground-truth FEM results (panel A). The lead-field results for the FEM method show a Coulomb-like, spherically-symmetric pattern for distances larger than, say, twice the electrode radius. However, closer to the disc electrode the highly conductive contact distorts this pattern, in particular in the lateral (*x*) direction, i.e., parallel to the electrode surface. The point-electrode MoI approximation implies almost a fully Coulombic lead field in the half-sphere *z*>0 (only the saline cover breaks the 1/*r* dependence corresponding to perfect spherical symmetry). As a consequence this approximation will predict too high lead-field values close the electrode, cf. panel C.

The disc-electrode MoI approximation (panel E) gives much more accurate predictions for the lead field (panel F). Panel B summarizes the relative errors, i.e., relative deviations from the ground-truth FEM results, in the predicted lead fields for the two MoI-approximations as functions of height above the electrode center for three different electrode radii: 5, 10 and 15 *μ*m. A first observation is that the relative lead-field error is generally much smaller for the disc-electrode MoI approximation. Further, for both MoI approximations the error is essentially only dependent on the distance measured in units of electrode radii. The small variation in error for these three electrode-size curves in panel B is due to the saline interface at 300 *μ*m and the finite number of sampling points in evaluating the sum in Eq. 22.

In panel B we further see that for distances larger than half the electrode radius, the deviation for the disc-electrode MoI approximation is less than 10 *%*. For distances larger than 3–4 times the electrode radius, this error is reduced to less than 1 *%*. While the calculated relative error values in panel F will depend somewhat on the mesh and the number of sampling points in Eq. 22, this 2D plot may serve as rule of thumb when considering the effects of the finite size of disc electrodes without resorting to FEM simulations. However, also the simple point-electrode MoI may work quite well: the deviation from the FEM results is seen in panel B to be less than 10 *%* for distances larger than two electrode radii, while it is less than 1 *%* for distances larger than about eight times the electrode radius.

### Electrically Anisotropic Brain Tissue

So far we have only considered brain tissue with isotropic electrical conductivity, i.e., the same conductivity in all directions. Anisotropic electrical conductivities have, however, been observed in frog cerebellum (Nicholson and Freeman 1975), guinea-pig hippocampus (Holsheimer 1987), and rat neocortex (Goto et al. 2010). In the rat somatosensory barrel cortex, Goto et al. (2010) found the conductivity in the depth direction, i.e., parallel to the long apical dendrites, to be up to 50 % larger than in the lateral directions. For the typical MEA set-up for cortical slice studies (Bakker et al. 2009) this would correspond to a larger conductivity in the *x*-direction than in the *y*- and *z*-directions, cf. Fig. 1.

**Fig. 6:**
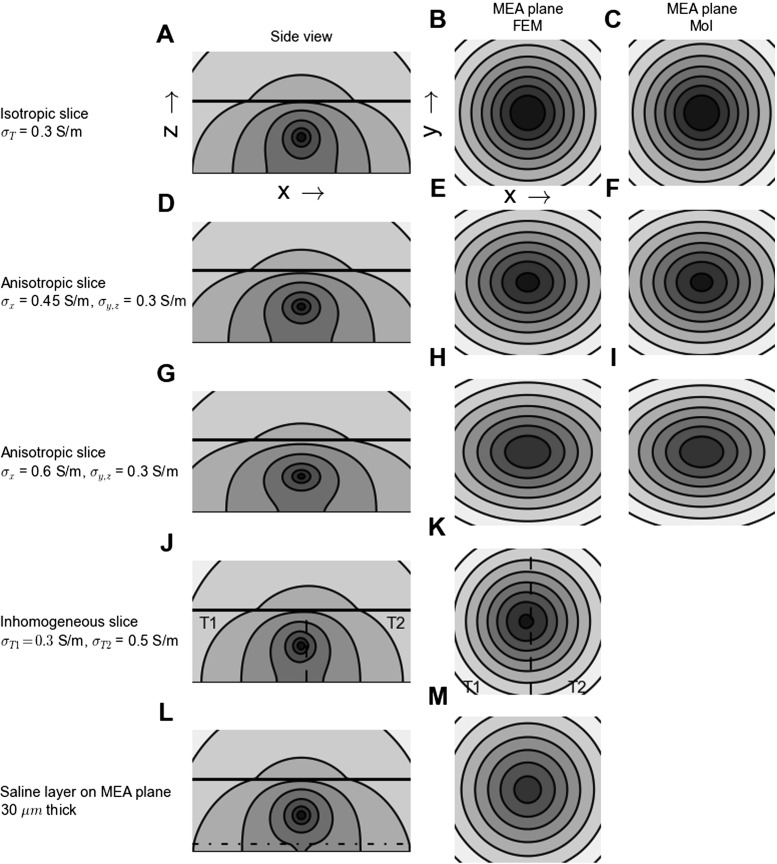
Effect of anisotropic and inhomogeneous electrical conductivities on electrical potentials. **A–C**: Electrical potential from a current point source positioned in the middle of a *h*=300 *μ*m thick brain slice sandwiched between a fully insulating MEA plane and an (infinitely thick) saline cover. The slice is isotropic and homogeneous with *σ*_*T*_ = 0.3 S/m and covers the entire ( *x*,*y*) plane. The saline cover has *σ*_*S*_ =1.5 S/m. Panel **A** shows a contour plot of the FEM potential in an 900 ×450 *μ*m section of ( *x*,*z*)-plane extending from *z*=0 (MEA plane) displaced 10 *μ*m in the *y*-direction from the current source. The interface between slice and saline is indicated by the black horizontal line. The contour plot is logarithmic, and the potential is reduced by 50 % between contour lines. Panels **B** and **C** show contour plots of the potential in a 1000 ×1000 *μ*m section of MEA-plane (( *x*,*y*)-plane for *z*=0) with the ( *x*,*y*)-position of the current source in the center of the square section for finite-element method, FEM (**B**) and method of images, MoI (**C**). The contour plots are logarithmic with the potential reduced by 25 % between contour lines. **D–F**: Same as panels **A–C** with moderate (50 %) anisotropy in the conductivity in the brain slice, i.e., a larger conductivity *σ*_*Tx*_=0.45 S/m in the *x*-direction than in the *y* and *z* directions, *σ*_*Ty*_=*σ*_*Tz*_=0.3 S/m **G–I**: Same as panels **A–C** with stronger (100 %) anisotropy in the conductivity in the brain slice, i.e., a larger conductivity *σ*_*Tx*_=0.6 S/m in the *x*-direction than in the *y* and *z* directions, *σ*_*Ty*_=*σ*_*Tz*_=0.3 S/m. **J–K**: Same as panels **A–B** with inhomogeneous conductivity in the slice, i.e., different conductivities on each side of the vertical dashed line. Electrical conductivities are *σ*_*T*1_=0.3 S/m (left side) and *σ*_*T*2_=0.5 S/m (right side). Current source is positioned on the left side (region 1), 20 *μ*m from the interface between tissue regions 1 and 2. **L–M**: Same as panels **A–B** with a 30 *μ*m thick saline layer replacing the brain tissue immediately on top of the insulating MEA surface

In Fig. 6 we illustrate the effects of such anisotropy on the spatial distribution of the electrical potential around a current point source positioned in the middle of a brain slice in our MEA set-up. As before the slice is *h*=300 *μ*m thick and sandwiched between a fully insulating MEA plane and an (infinitely thick) saline cover. Panels A–C show the reference case with isotropic conductivity, while panels D–F show an anisotropic example with 50 % larger conductivity in the *x*-direction than in the other directions, similar to what was reported in Goto et al. (2010). To highlight qualitative effects of such anisotropy further, we show in panels G–I corresponding results for an exaggerated anisotropy, i.e., a factor two larger conductivity in the *x*-direction. The most prominent feature of panels D–F, seen to be even more pronounced in panels G–I, is the ellipsoidal shape of the predicted electrical potentials so that the recorded MEA potential decays less steeply in the high-conductivity *x*-direction than in the low-conductivity *y*-direction.

**Fig. 7:**
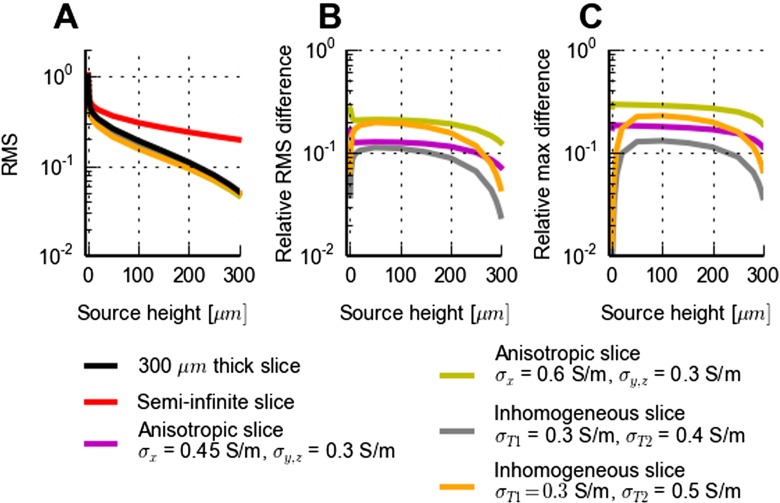
Effects of anisotropy and inhomogeneity in electrical conductivity on MEA lead field. **A**: FEM predictions of RMS (root-mean-square; see Methods, Eq. 21) of lead fields of MEA potentials for different electrical conductivity scenarios for a 300 *μ*m thick slice of brain tissue covered with saline: homogeneous and isotropic (*σ*_*T*_=0.3 S/m, black), homogeneous and anisotropic (*σ*_*Tx*_=0.45 S/m, *σ*_*Ty*_= *σ*_*Tz*_=0.3 S/m, purple; *σ*_*Tx*_=0.6 S/m, *σ*_*Ty*_= *σ*_*Tz*_=0.3 S/m, green), inhomogeneous and isotropic ( *σ*_*T*1_=0.3 S/m, *σ*_*T*2_=0.4 S/m, grey; *σ*_*T*1_=0.3 S/m, *σ*_*T*2_=0.5 S/m, orange), see Fig. 6 for illustration. Results for semi-infinite homogeneous and isotropic slice ( *σ*_*T*_=0.3 S/m) is in red. The RMS signal is found by averaging the MEA potentials over 101 ×101 equidistant points on a 1 mm by 1 mm square grid in the MEA plane positioned with the center point under the current source, cf. Eq. 21. **B**: Illustration of difference between predictions of lead field for anisotropic or inhomogeneous electrical conductivities in **A** and predictions assuming homogeneous and isotropic conductivity ( *σ*_*T*_=0.3 S/m). Depicted relative RMS difference corresponds to the RMS of the difference divided by the RMS of the result for homogeneous and isotropic conductivity (black line in **A**).**C**: Alternative illustration of difference between anisotropic or inhomogeneous electrical conductivities and homogeneous & isotropic reference case. Here the maximum deviation between the lead-field predictions for the various examples and the reference case, normalized by the maximum lead-field value for the reference case (which corresponds to the electrode positioned immediately below the point source), is shown as a function of point-source height

The effects of anisotropic conductivity are further explored in Fig. 7. Panel A shows the contact-averaged RMS signal (see Methods, Eq. 21) for the MEA lead field as a function of the current source height above the MEA plane. This reveals on average relatively modest deviations of the lead field for the two anisotropic examples ( *σ*_*Tx*_=0.45 S/m, *σ*_*Ty*_= *σ*_*Tz*_=0.3 S/m and *σ*_*Tx*_=0.6 S/m, *σ*_*Ty*_= *σ*_*Tz*_=0.3 S/m) compared to the isotropic reference case ( *σ*_*T*_=0.3 S/m), at least compared to the effect of adding a saline cover (panel A). To highlight the differences in the anisotropic cases, panel B shows the relative RMS difference between each anisotropic example and the isotropic reference, normalized by the RMS of the reference. For the example with the strongest anisotropy ( *σ*_*Tx*_/*σ*_*Ty*,*z*_=2), the relative RMS difference is seen to be around 20 % for all source heights. For the example with *σ*_*Tx*_/*σ*_*Ty*,*z*_=0.45/0.3=1.5, similar to what has been seen in neocortical experiments (Goto et al. 2010), the RMS difference is reduced to about 10 %. Note also that not all of this deviation from the isotropic result stems from the anisotropy itself as also the ‘average’ conductivity is altered. To complement the RMS measure which averages the deviations across many contacts, we show in panel C another deviation measure from the same data, i.e., the maximum deviation between the lead-field predictions for the various examples and the reference case, normalized by the maximum lead-field value for the reference case (corresponding to the electrode positioned immediately below the point source). Qualitatively, these deviation curves look similar to the results for the RMS measure in panel B.

**Fig. 8:**
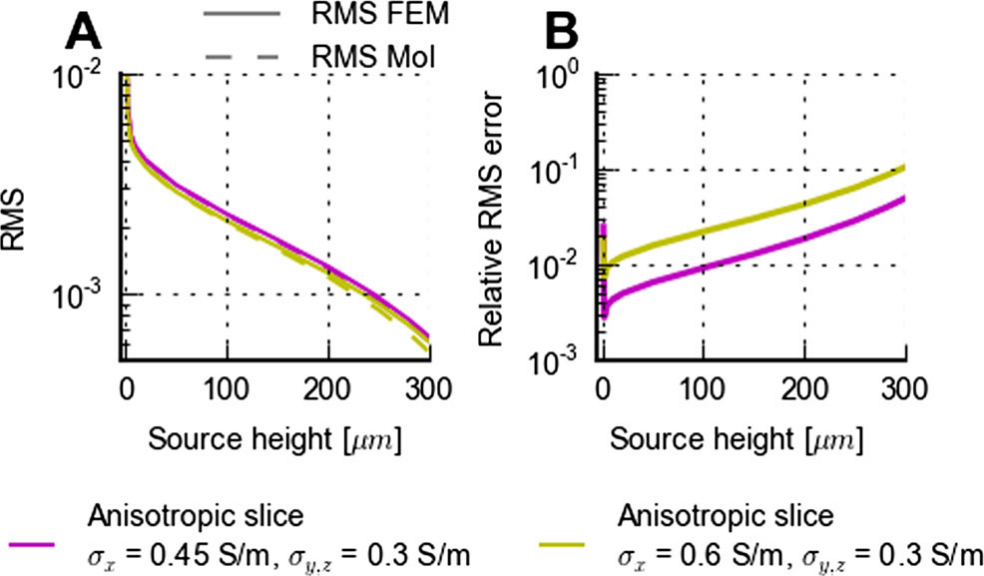
Applicability of MoI approximation for electrically anisotropic brain slices. **A**: FEM and MoI predictions (14) of RMS (root-mean-square; see Methods, Eq. 21) of lead fields of MEA potentials for a 300 *μ*m thick slice of electrically anisotropic brain tissue: *σ*_*Tx*_=0.45 S/m, *σ*_*Ty*_= *σ*_*Tz*_=0.3 S/m, purple; *σ*_*Tx*_=0.6 S/m, *σ*_*Ty*_= *σ*_*Tz*_=0.3 S/m, green. The RMS signal is found by averaging the MEA potentials over 101 ×101 equidistant points on a 1 mm by 1 mm square grid in the MEA plane positioned with the center point under the current source, cf. (21). **B**: Illustration of difference between MoI and FEM predictions of lead field for anisotropic electrical conductivities in **A**. Depicted relative RMS difference corresponds to the RMS of the difference divided by the RMS of the FEM prediction

A visual comparison between MEA potentials predicted by the anisotropic MoI approximation (panels F and I, Eq. 14) and FEM (panels E and H) in Fig. 6, reveals apparently identical results. Thus for the particular example in this figure with the source positioned in the center of the slice, the inherent error in the anisotropic MoI approximation of assuming the same anisotropy structure in saline as in the slice, is seen to be small. However, this error is expected to be larger for current sources placed closer to the tissue-saline interface. This is indeed confirmed by the numerical results shown in Fig. 8 where the relative MoI RMS error, i.e., relative deviation between MoI and FEM results, is seen to increase as the source height approaches 300 *μ*m, i.e., the interface with saline. For the example with the largest anisotropy, i.e., twice as large conductivity in one direction as the two others, the relative RMS error is seen in Fig. 8B to be between 1 and 10 %.

### Inhomogeneous Brain Tissue

The electrical conductivity is not fully homogeneous across brain tissue. White matter is, for example, known to have a lower electrical conductivity than grey matter (Nunez and Srinivasan 2006; Logothetis et al. 2007), and inhomogeneous conductivities across layers have been measured both in hippocampus (López-Aguado et al. 2001) and in neocortex (Goto et al. 2010). In neocortex the inhomogeneity appears to be modest, however, maybe on the order of 10–20 % or less, cf. Table 5 in Goto et al. (2010). In Figure 6J–K we show results for a situation with a much exaggerated inhomogeneity where a current source is placed within a slice with *σ*_*T*1_=0.3 S/m next to a slab of tissue with a 67 % higher conductivity, i.e., *σ*_*T*2_=0.5 S/m. As apparent both in panels J and K, the neighboring high-conductivity slab (region 2) visibly distorts the electrical potential generated by the current source in the low-conductivity slab (region 1). This effect is further elucidated in Fig. 7 where panel A shows, as for the above examples of anisotropic conductivity, relatively modest deviations of the RMS signal, compared to the homogeneous and isotropic reference case, for the two inhomogeneous examples considered. Panel B shows that the relative RMS difference between the homogeneous reference case and the two-slab inhomogeneous case with *σ*_*T*1_=0.3 S/m and *σ*_*T*2_=0.5 S/m is always 20 % or less. For the less inhomogeneous situation with *σ*_*T*1_=0.3 S/m and *σ*_*T*2_=0.4 S/m, this relative difference is typically less than 10 %. Again, the results for the alternative ‘relative maximum’ deviation measure shown in panel C are qualitatively similar.

### Effect of Saline Cover for Finite Slice Thickness

**Fig. 9:**
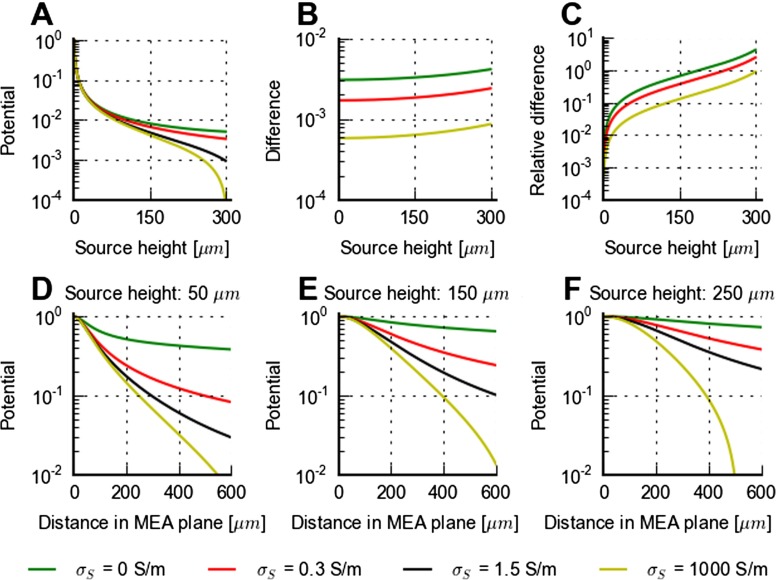
Effects of saline bath on MEA lead field. **A**: MoI predictions of MEA potential for a 300 *μ*m thick slice of brain tissue ( *σ*_*T*_=0.3 S/m) covered with liquids with different electrical conductivities: (i) insulating, e.g., oil ( *σ*_*S*_=0), (ii) tissue-like ( *σ*_*S*_=0.3 S/m), (iii) default saline ( *σ*_*S*_=1.5 S/m), (iv) ultra-conductive, i.e., essentially metallic ( *σ*_*S*_=1000 S/m). The depicted potentials, essentially corresponding to the lead field, correspond to recordings for a point-electrode positioned in the MEA plane directly below the current source. Potentials are normalized to the value found for a source height of 1 *μ*m. While results are shown for the point-electrode MoI approximation (8), essentially identical results are found with FEM. **B**: Magnitude of difference between MEA potentials in **A** and reference case with (default) saline cover ( *σ*_*S*_=1.5 S/m) **C**: Relative difference between MEA potentials in **A** and reference case with (default) saline cover ( *σ*_*S*_=1.5 S/m), i.e., difference in **B** divided by the potential predicted for the reference case. **D–F**: MoI predictions of MEA potentials for the same situations as in **A** with laterally displaced current sources for fixed source heights of *z*=50 *μ*m (panel **D**), *z*=150 *μ*m (panel **E**), and *z*=250 *μ*m (panel **F**), respectively. Here the potentials are normalized to the value found for zero lateral distance, i.e., current source positioned directly above the MEA electrode

As seen in Fig. 4, the high conductivity of the saline covering the brain slice may substantially affect the electrical potential compared to the (hypothetical) situation with an infinitely thick slice (or, alternatively, a saline cover with a low salt concentration so that the conductivity is reduced to the value within the brain slice). The example depicted in Fig. 4 corresponds to a current source positioned in the middle of the 300 *μ*m thick slice (*z*=150 *μ*m), and the saline-cover effect will be larger when the current source is positioned closer to the tissue-saline interface. This position dependence is shown quantitatively in Fig. 9 for a set of different (hypothetical) bath conductivities: (i) insulating, e.g., oil ( *σ*_*S*_=0), (ii) tissue-like ( *σ*_*S*_=0.3 S/m), (iii) default saline ( *σ*_*S*_=1.5 S/m), (iv) ultra-conductive, i.e., essentially metallic ( *σ*_*S*_=1000 S/m). Figure 9A shows that the electrical potential close to the bath surface is strongly affected by the conductivity of the bath. For the ultra-conductive case ( *σ*_*S*_=1000 S/m) the bath essentially short-circuits the electrical potential at the tissue-bath interface. Further, even for our default saline cover ( *σ*_*S*_=1.5 S/m), the potential close to the bath surface is reduced by a factor of ten or so from the case with tissue-like conductivity ( *σ*_*S*_=0.3 S/m) or an insulating medium ( *σ*_*S*_=0).

The effects of changing the electrical conductivity of the bath covering the brain slice are further illustrated in Fig. 9B–C showing the difference between predicted MEA potentials for the various alternatives considered in panel A and the reference case with our default saline cover (case (iii)). While the differences are small compared to the absolute magnitude of the potentials for source heights less than about 75 *μ*m, this is not so for sources close to the tissue-bath interface at 300 *μ*m. Here, a comparatively insulating bath with tissue-like conductivity ( *σ*_*S*_=0.3 S/m), and even more for a fully insulating bath ( *σ*_*S*_=0), gives a substantially larger potential compared to the reference case. An ultra-conductive bath ( *σ*_*S*_=1000 S/m), on the other hand, would give a smaller potential for sources placed close to the tissue-bath interface. However, the difference from the reference case is smaller, so our reference situation with a saline cover with *σ*_*S*_=1.5 S/m is closer to the short-circuit limit ($\sigma _{S} \rightarrow \infty $) than the insulating limit ( *σ*_*S*_=0).

Qualitatively, the above findings on the role of the bath, and the bath conductivity in particular, also apply to the more general case when the current source is displaced laterally compared to the recording MEA electrode contact. This is illustrated in panels D–F in Fig. 9. The general trend is that while the overall potential reduces sharply with increasing source heights (cf. panel A), the slope of the decay of the MEA potential in the lateral direction is smaller. For example, panel D shows that for the reference case ( *σ*_*S*_=1.5 S/m) and a source height of *z*=50 *μ*m, the potential is reduced to less than 5 % of the on-center value for a lateral distance of 600 *μ*m. For a source height of *z*=250 *μ*m (panel F) the relative potential is only reduced to about 20 % at the same lateral distance. Thus, the saline cover both reduces the overall amplitude of the recorded MEA potentials and makes the signal more ‘local’ in the lateral directions.

### Effect from Putative Saline Layer at MEA-Slice Interface

The detailed electrical properties of the interfacial region between the MEA chip and the brain slices are largely unknown. It is, for example, conceivable that a thin saline layer covers the MEA chip, and such a high-conductive layer may distort the potentials recorded at the MEA electrodes. Here we explore putative effects of such a saline interface layer. As information about typical layer thicknesses is lacking, we somewhat arbitrarily chose to consider layer thicknesses of 10 and 30 *μ*m. While in particular 30 *μ*m expectedly is a gross overestimation of the typical case, it serves to highlight the qualitative effects of such an interface layer on the MEA potentials, i.e., the MEA lead fields.

**Fig. 10:**
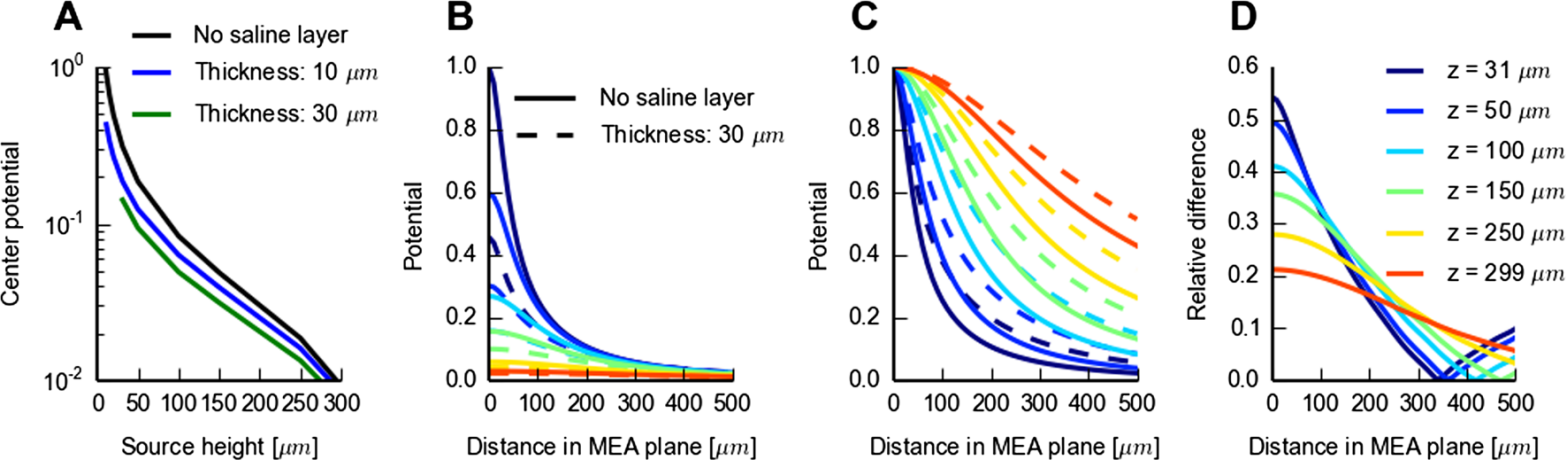
Effect of saline layer at MEA-tissue interface. **A**: FEM predictions of MEA center potential (current source directly above electrode) for an isotropic and homogeneous slice of brain tissue ( *σ*_*T*_=0.3 S/m), covered with saline, where the bottom part of the slice is replaced by a thin saline layer (10 and 30 *μ*m, respectively) so that the total thickness (MEA-tissue saline interface layer + tissue) is kept at 300 *μ*m. The saline conductivity is *σ*_*S*_=1.5 S/m both in the interface layer and in the slice-covering bath. The depicted potentials, corresponding to the MEA lead fields, are normalized to the value found for a source height of 11 *μ*m for the reference case (no interface layer). The curves for the 10 and 30 *μ*m saline-layer cases start at 11 and 31 *μ*m, respectively (as current sources can only be within the brain tissue slice). **B**: FEM predictions of MEA potentials as in **A** for laterally displaced current sources for a set of different fixed source heights. Only results for a 30 *μ*m thick interface layer and no-layer reference case are shown. **C**: Results in B normalized by the values obtained when the point source is positioned above the MEA electrode contact. **D**: Relative difference between situations with and without saline interface layer in **B**

Panels L and M of Fig. 6 illustrate the effect of replacing the bottom 30 *μ*m of a 300 *μ*m thick brain slice with the much more conductive saline. When comparing with the reference case in panels A and B, we observe that the main effect of the saline layer seems to be a reduction of the overall MEA potential amplitude, in particular for the electrodes positioned beneath the current source, i.e., small lateral displacement. As shown in Fig. 10 the reduction of the MEA potential by the saline layer is largest for current sources placed (i) close to the MEA plane and (ii) directly above the MEA electrode. For example, in panel D it is seen that while the relative reduction of the MEA potential for a centered electrode is more than 50 % for a current source placed adjacent to the saline interface layer ( *z*=31*μ*m), the corresponding reduction for a current source placed adjacent to the tissue-bath interface ( *z*=299*μ*m) is less than 25 %. And both these numbers for the relative reduction decrease when the current source is shifted laterally away from the recording electrode (for lateral distances less than about 350 *μ*m, cf. panel D in Fig. 10). Thus, unlike the saline cover, a saline layer at the MEA-slice interface will make the recorded MEA potentials less ‘local’ in the lateral directions, cf. panel C.

### Forward-Modelling of Spikes

**Fig. 11:**
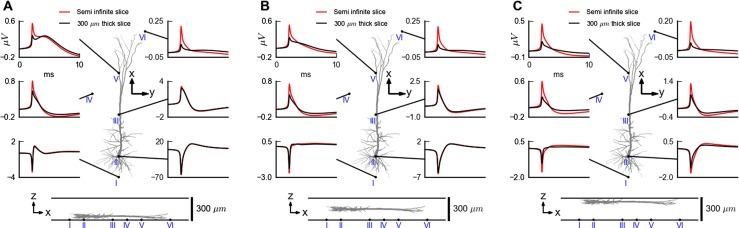
Illustration of effect of saline bath on recorded neural spike. Extracellular spike from a model layer-5 pyramidal cell (Hay et al. 2011) positioned with its soma 50 *μ*m (**A**), 150 *μ*m (**B**) and 250 *μ*m (**c**), respectively, above the MEA chip with the apical dendrite oriented in parallel to the MEA plane. Results from two cases are shown: (i) Reference case with 300 *μ*m thick brain slice ( *σ*_*T*_ = 0.3 S/m) covered with saline ( *σ*_*S*_ = 1.5 S/m) and (ii) a semi-infinite slice with *σ*_*T*_ = 0.3 S/m throughout. Predicted spikes at six different positions in the MEA plane are shown, cf. bottom illustrations. All simulations are done using MoI with the line source equation (9). In the final modelling step where the extracelluar potential is calculated based on transmembrane currents, the cell has been squeezed by a factor of two in the *z*-direction to keep the entire cell within the slice for all positions

One application of MEAs is the monitoring of spiking activity in acute retinal (Segev et al. 2004; Zeck et al. 2011) and brain slices (Egert et al. 2002; Frey et al. 2009; Hierlemann et al. 2011). Above we saw that the saline bath covering the slice may have substantial effects on the recorded MEA potential, and in Fig. 11 we explore the effects this may have on a recorded neural spike. In this example a neocortical layer-5 pyramidal model cell (Hay et al. 2011) is positioned inside a 300 *μ*m thick brain slice with the apical dendrite oriented parallel to the MEA plane. Example results are shown for three different choices of soma heights: close to the MEA plane (*z*=50 *μ*m, panel A), in the middle of the slice (*z*=150 *μ*m, panel B), and close to the saline interface (*z*=250 *μ*m, panel C).

A first observation is that for the two first situations depicted in panels A and B, the largest signals are seen for the electrode placed immediately below the soma. In particular, for the neuron placed closest to the MEA plane (panel A), the peak-to-peak amplitude of the spike is seen to be about 100 *μ*V for this soma-centered electrode, similar to what was found for a cerebellar Purkinje cell placed 40 *μ*m above the MEA chip in Frey et al. (2009). This observation is readily understood on the basis of the biophysical properties of the neuron: during spiking the strongest transmembrane currents go through the soma and proximal dendrites, and in accordance with the fundamental forward formula in Eq. 1 the largest spikes will generally be seen for electrodes positioned close to the soma (Hold and Koch 1999; Gold et al. 2006, 2007; Pettersen et al. 2008).

A comparison of the spike waveform recorded by the soma-centered electrode between the reference case ( *σ*_*T*_ = 0.3 S/m and saline cover with *σ*_*S*_ = 1.5 S/m) and the case without a conductivity jump (semi-infinite slice with *σ*_*T*_ = 0.3 S/m throughout) reveals negligible saline-bath effects. The effect of the saline cover on the spike waveform for the same electrode is as expected more pronounced when the neuron is placed close to the saline interface (panel C), but this is experimentally less important as the MEA potential in any case is substantially reduced with a peak-to-peak amplitude of only about 2 *μ*V. For all soma heights, much larger effects of the saline bath are seen for MEA electrodes positioned below the distal apical part. However, also here the amplitudes of the potentials are generally much reduced compared to the potential at the soma-centered electrode.

When exploring the differences between the reference saline-cover case and the semi-infinite slice case in Fig. 11 further, we see that the saline cover generally reduces the recorded potentials most at the electrodes furthest away from the soma. This is readily understood on the basis of the findings in Fig. 9 showing that the highly conductive saline cover reduces the recorded potential most for electrodes displaced farthest away laterally compared to the position of the current source. Consequently, as the dominant current sources are close to the soma during an action potential, the reduction of the recorded spike potential will be largest for the electrodes positioned far away from the soma, cf. Fig. 11. Thus the saline cover makes the spike more ‘local’ in the lateral direction as the spike potentials spread shorter in the lateral direction than it would have in the analogous semi-infinite slice.

**Fig. 12:**
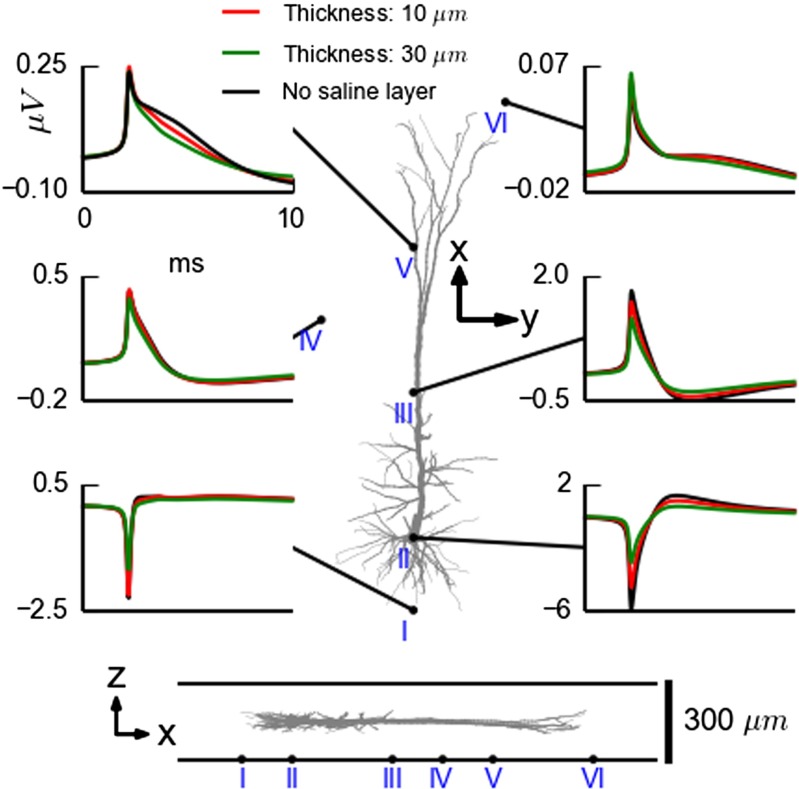
Illustration of effect of thin saline layer covering MEA chip on recorded neural spike. Extracellular spike from a model layer-5 pyramidal cell (Hay et al. 2011) positioned with its soma 150 *μ*m (*center*) above the MEA chip with the apical dendrite oriented in parallel to the MEA plane. Results from three cases are shown: no saline layer between the MEA and slice (*black*), and saline layers of different thicknesses (*blue*: 10 *μ*m, *green* 30 *μ*m) keeping the total saline layer + slice thickness constant at 300 *μ*m, cf. Fig. 10. Predicted spikes at six different positions in the MEA plane are shown, cf. bottom illustrations. All simulations are done using FEM. The cell has been squeezed by a factor of two in the *z*-direction after the neural simulation to keep the entire cell within the slice

The effect of a saline layer between the MEA and the brain slice on the predicted waveform of a spike is shown in Fig. 12. Notice that for the electrode closest to the soma (position II) where the spike is largest, the saline layer between the MEA chip and slice strongly reduces the spike amplitude, from 7.2 *μ*V for the peak-to-peak amplitude for the no-layer reference case to 5.5 *μ*V and 3.6 *μ*V for the cases with 10 *μ*m and 30 *μ*m saline layers, respectively. For the electrodes far away from the soma (e.g., at positions IV and VI), on the other hand, there are only minor effects on the amplitude from a thin saline chip-slice interface layer.

### Forward-Modelling of LFP from Cortical Network

**Fig. 13:**
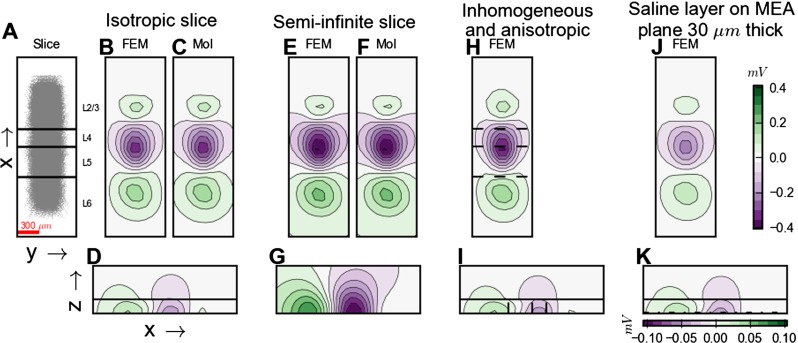
Snapshot of LFP generated by neural network activity. **A**: Illustration of geometrical placement of network model on MEA chip. **B–C**: Snapshot image of electrical potential (LFP) generated by the Traub network model centered in the middle of a *h*=300 *μ*m thick brain slice sandwiched between a fully insulating MEA plane and and an infinitely thick saline cover. The slice is isotropic and homogeneous with *σ*_*T*_=0.3 S/m and covers the entire ( *x*,*y*) plane. The saline cover has *σ*_*S*_=1.5 S/m. Panels **B** and **C** show contour plots of the potential in a 3000 × 1000 *μ*m section of MEA-plane (( *x*,*y*)-plane for *z*=0) for FEM (**B**) and the method of images, MoI (**C**). Panel **D** shows a contour plot of the FEM potential for the same situation as in **B** in a 3000 ±1000 *μ*m section of ( *x*,*z*)-plane, passing through the center of the model network in the *y*-direction, extending from *z*=0 (MEA plane). The interface between the slice and saline is indicated by the black horizontal line. The isopotential lines in the contour plots are linearly spaced and centered around zero (16 levels between -1 and 1, inclusive). **E–G**: Same as panels **B–D** for a corresponding semi-infinite slice (i.e., the same electrical properties in the saline cover as in the slice) extending from the MEA chip. **H, I**: Same as panels **B, D** with anisotropic and inhomogeneous electrical conductivity following the experimental findings for rat somatosensory cortex (Goto et al. 2010), see Table 2. Vertical dashed lines in slice depict interfaces between cortical layers. **J, K**: Same as panels **B, D** with a 30 *μ*m thick saline layer replacing the brain tissue immediately on top of the insulating MEA surface

In Fig. 13 we show a snapshot of the local field potential generated by Traub’s cortical network model for various assumptions regarding MEA set-up and recording conditions, both in the MEA plane (top row) and in a side view (bottom row). We use here the conventional term local field potential (LFP) as the signal is dominated by low frequency contributions from synaptic and associated return currents, although it has not been filtered. At this particular time the LFP pattern is dominated by a negative LFP in layers 4 and 5, surrounded by positive LFP above and below. Comparison of the results for the reference case (300 *μ*m slice with saline cover; panels B–D) with the case without a conductivity jump, i.e., semi-infinite slice (panels E–F), shows substantial differences. For the potentials recorded in the MEA plane (panels B–C, E–F) we observe in particular that the saline bath reduces the amplitudes of the recorded potentials. For completeness we also demonstrate the anticipated close agreement between our numerically comprehensive FEM results (panels B and E) with corresponding MoI results (panels C and F).

**Fig. 14:**
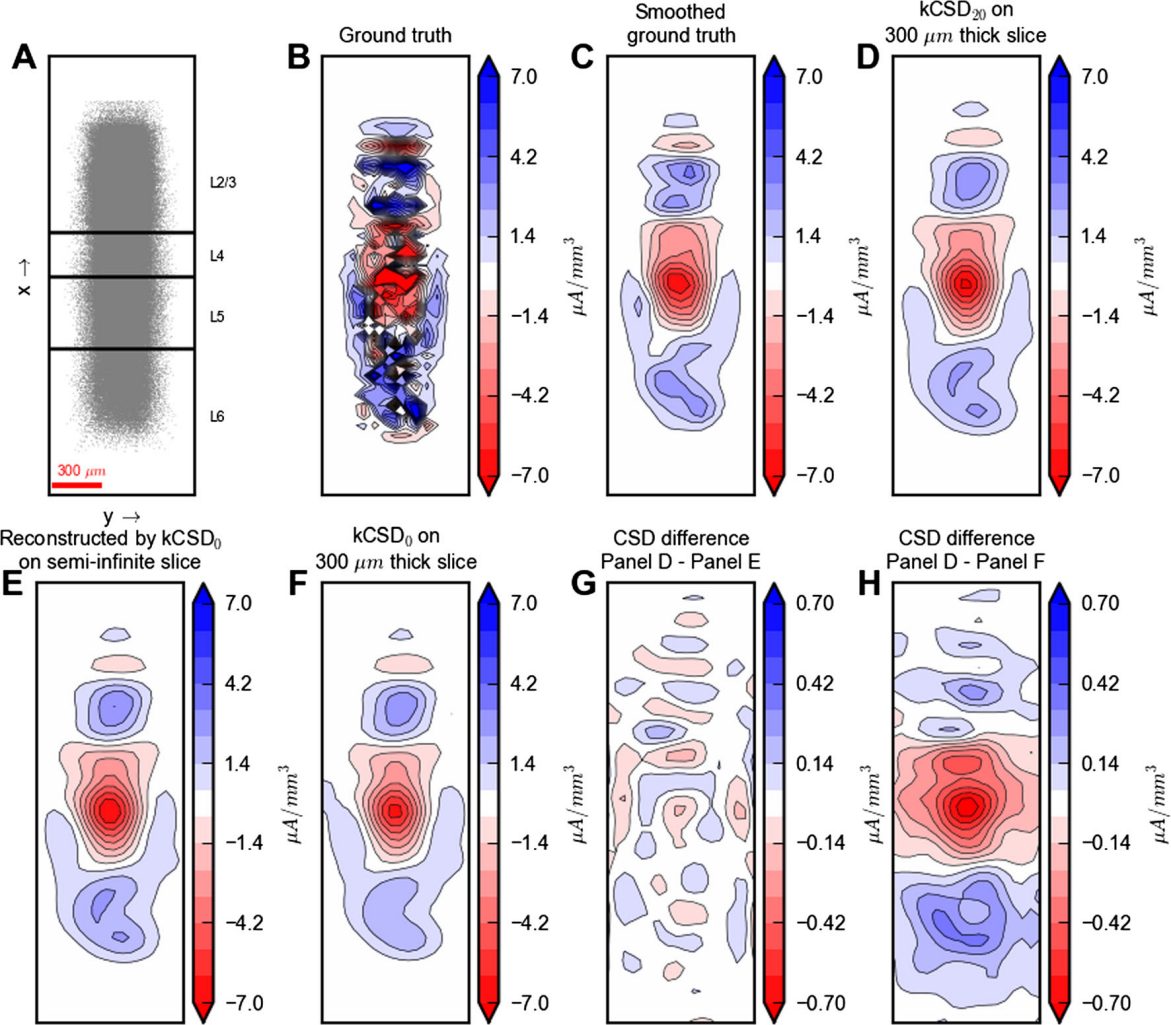
Reconstruction of CSD from MEA potential recordings. **A**: Top view of the model set-up. **B**: Ground-truth CSD distribution from Traub’s cortical network model. **C**: Ground-truth CSD (**B**) smoothed with a Gaussian kernel of *σ*≈75*μ* *m*. **D**: Reconstructed CSD assuming saline cover both in the forward modelling of LFP (forward model), and in the kCSD method. **E**: Reconstructed CSD assuming no saline cover in either calculation of LFP or the kCSD method. **F**: Reconstructed CSD assuming saline cover in the forward modelling of LFP (forward model), but neglecting the saline cover correction in the kCSD method. **G**: Difference between **D** and **E**. **H**: Difference between **D** and **F**

Next, panels H, I show results as for the reference-case situation in panels B, D except that now the brain slice is assumed to have anisotropic and inhomogeneous electrical conductivity in line with the experimental findings for rat somatosensory cortex in Goto et al. (2010), see Table 2. The deviations from the isotropic and homogeneous reference case in panel B,D are very small, only a slight increase in potential amplitude, mainly due to the imposed anisotropy, can be seen.

In the final panels in Fig. 13 (panels J, K) we show the effects of having a putative thin saline layer between the brain slice and the MEA chip. As expected from our previous findings summarized in Fig. 10, the main effect of such a saline layer is a reduction of the amplitude of the recorded MEA potentials.

### CSD Analysis of MEA Potentials

As shown above, the saline bath covering the brain slice may have non-negligible effects on the LFP potentials recorded by the MEA electrodes. One may thus expect that ignoring these effects may induce errors in current-source densities (CSDs) estimated from MEA recordings (Łęski et al. 2011). Since in the more recent CSD estimation methods based on inversion of forward models, like the iCSD (Pettersen et al. 2006; Lęski et al. 2007, 2011) and kCSD (Potworowski et al. 2012), the saline effects may be explicitly accounted for, we next investigate their importance for the estimated CSD profiles.

In Fig. 14 we contrast ground-truth current sources from the model with different CSD reconstructions. The MEA LFP data correspond to the same time point as above, i.e., the data depicted in Fig. 13. Clearly, the spatial complexity of the model (panel A), results in a complicated microscopic distribution of transmembrane currents (panel B). The distance between the MEA metal microelectrode contacts sets a lower limit on the spatial scale of CSD which can be resolved. Here, where the interelectrode distance is set to be about 100 *μ*m, the microscopic details of the ground-truth CSD pattern are beyond reach for any CSD estimation method. Only a coarse-grained CSD can realistically be obtained. In panel C we show the data from panel B spatially smoothed with a Gaussian kernel of width *σ*=1.1 times the voxel size, which here is 67 *μ* *m*. The size of this smoothing kernel has been adapted to qualitatively match the reconstructed CSD (e.g., panel D, E, F) and reflects the coarser spatial scale set by the interelectrode distance in the MEA array (here 103 *μ*m in the *x*-direction and 111 *μ*m in the *y*-direction, i.e., 30 by 10 electrodes spanning the area of 3000 × 1000 *μ* *m* ^2^ (Łęski et al. 2011)).

Panel D shows the CSD estimated using the kCSD _20_ method based on the three-layer MoI formula in Eq. (8). The potentials used for the reconstruction were computed in the MEA plane for the saline-cover reference case, i.e., data shown in panel B in Fig. 13. As we can see, the recovered CSD pattern very closely matches the spatially-smoothed, ground-truth CSD pattern shown in panel C, testifying to the accuracy of the MoI-based kCSD method.

Panel E correspondingly shows the estimated CSD pattern resulting from applying the (no-saline) kCSD _0_ method on the corresponding (no-saline) MEA LFP i.e., data shown in panel E in Fig. 13. Here the forward model of Eq. 8 is used without the series sum, i.e., with *W*_*TS*_=0. Physically, this corresponds to neglecting corrections due to the different electrical conductivities of slice and saline. As expected, given that the appropriate forward model is built into the CSD estimator, the estimated CSD pattern is seen to be essentially identical to the estimated CSD pattern for the saline-cover case in panel D. The small differences between the two CSD estimates are shown in panel G (note different color scale from panels D–F).

A natural question regards the effect of the saline on the CSD reconstruction, that is, how big is the error we make if we neglect the saline cover when constructing the CSD estimator, but nevertheless apply it on the saline-cover MEA potentials? Panel F shows the CSD reconstructed from the same potentials as in panel D, but by use of the (no-saline) kCSD _0_ method instead. Visual comparison between the estimated CSDs in panels D and F shows that the deviations are small. This is further illustrated by the plot of the differences in the two CSD estimates in panel H revealing that the differences between these CSD estimates are on the order of 10 %. So while the saline cover has a non-negligible effect on the recorded MEA potentials, its practical effect on the CSD estimator is relatively small.

The observation that the saline correction can be neglected in CSD estimation can be understood by detailed inspection of the underlying physical forward-modelling formulas. According to the MoI forward-model formula in Eq. 8, the LFP in the saline-cover reference case can be considered to be built up from two contributions: the first term corresponding to the semi-infinite slice situation and the correction term resulting from the infinite series of image current sources. It turns out, as shown below, that the correction term is negligible so that in practice one may neglect the saline interface in the forward model when constructing the CSD estimator.

To demonstrate this important point it is easier to consider the ‘traditional’ CSD method (Nicholson and Freeman 1975) rather than the kCSD method. In the traditional method the CSD estimator is essentially given by the two-dimensional Laplace operator $\nabla ^{2} = {\partial _{x}^{2}} + {\partial _{y}^{2}}$. Consider a single current source positioned at (0,0,*z*) inside the brain slice. The closest virtual image source, corresponding to the first term in the series in the MoI formula in Eq. 8, will then be positioned at (0,0,2*h*−*z*).

If we denote the potential at the MEA plane by *ϕ*_*P*_ and *ϕ*_*I*_ for the principal and first image sources, respectively, we find the following ratio of the potentials stemming from the image vs. principal sources:

23 $$ \left |\frac{\phi_{I}}{\phi_{P}}\right |_{x,y=0} = \left | W_{TS}\frac{z}{2h - z} \right |,  $$

and, by applying the two-dimensional Laplace operator, we find the ratio of contributions to the estimated CSD:

24 $$ \left |\frac{\nabla^{2}\phi_{I}}{\nabla^{2}\phi_{P}}\right |_{x,y=0} = \left | W_{TS}\frac{z^{3}}{(2h-z)^{3}} \right |,  $$

where for reasons of transparency of the argument we consider the same lateral position (*x*,*y*) as that of the current source. For a source in the middle of the slice, *z* = 150 *μ*m, with *σ*_*T*_ = 0.3 S/m and *σ*_*S*_ = 1.5 S/m, so that *W*_*TS*_=−2/3 (cf. Eq. 6), this implies

25 $$\begin{array}{@{}rcl@{}} \left |\frac{\phi_{I}}{\phi_{P}}\right |_{x,y=0;z=150 \mu m} &=& \frac{2}{9}, \end{array} $$

26 $$\begin{array}{@{}rcl@{}} \left |\frac{\nabla^{2}\phi_{I}}{\nabla^{2}\phi_{P}}\right |_{x,y=0;z=150 \mu m} &=& \frac{2}{81}. \end{array} $$

We thus find that relative correction to the CSD estimate from the first image source, Eq. 26, is an order of magnitude smaller than the corresponding relative correction to the potential itself, Eq. 25. The physical effect underlying this observation is that the CSD essentially is given by the curvature of the LFP, and this curvature decays faster with distance from the current source than the LFP itself.

## Discussion

While microelectrode arrays have long been used to record neuronal activity in *in vitro* brain slices with high spatial and temporal resolution (Taketani and Baudry 2006), the analysis of the recorded MEA potentials has generally been mainly qualitative. Here we have used a well-established biophysical forward-modelling formalism based on the finite element method (FEM) (Larson and Bengzon 2013) to establish a quantitatively accurate link between neural activity in the slice and potentials recorded in the MEA set-up, i.e., to allow for ‘virtual measurements’ in simulations of neural activity. This forward model is not only essential for the proper neurobiological interpretation of MEA potentials, it also allows for construction and verification of new analysis methods, exemplified by the CSD-estimation method investigated here (Pettersen et al. 2012; Einevoll et al. 2013a). As the FEM approach is computationally demanding, we have also explored a simpler method based on the method of images (MoI) from electrostatics (Jackson 1998) which allows for computation of MEA potentials by formulas analogous to what is used for homogeneous volume conductors (cf. Eq. 1). It turns out that MoI can be used in most situations of practical interest, and the Python software package ViMEAPy (*Virtual MEA signals in Python*) is made freely available to facilitate such forward-modelling of MEA potentials from simplified or biophysically detailed multicompartmental neurons.

Explicit MoI-based forward-model expressions linking a current source in the slice to MEA potentials can be derived assuming (i) idealized point electrodes, (ii) a planar and electrically homogeneous brain slice placed between a (here fully insulating) MEA chip, and (iii) an infinitely thick slab of homogeneous covering material (here saline). The formulas for the case where both the slice and the cover are electrically isotropic are given in Eqs. 8 and 9 for the point-source and line-source approximations, respectively. The corresponding point-source formula for the case where the slice and cover are assumed electrically anisotropic, yet with the same ratios between conductivities in the different directions, is given in Eq. 14. A more relevant situation for the present application is the case where an electrically anisotropic brain slice is covered by electrically isotropic saline. While the same analytical approach cannot be applied in this case, we found that the approximation of assuming the same anisotropy structure in the saline as in the slice, introduces negligible errors except maybe for sources positioned very close to the slice-saline interface, cf. Fig. 8.

We found that the saline cover may substantially reduce the amplitude of recorded MEA potential from a current source (compared to the hypothetical case with a semi-infinitely thick slice). This reduction is, not surprisingly, largest for current sources positioned close to the slice-saline interface, cf. Fig. 9A-C. This dampening effect is particularly pronounced when the potential is recorded by a contact which is laterally displaced from the current source, cf. Fig. 9D-F. Thus, in addition to reducing the overall amplitude of the recorded MEA potentials, the saline cover also makes the signal more ‘local’ in the lateral directions. In contrast, a saline layer at the MEA-slice interface will make the recorded potentials less local in the lateral directions, cf. Fig. 10.

Even with exaggerated assumed anisotropies and inhomogeneities in the electrical conductivity compared to what has been measured in cortex (Goto et al. 2010), the effects from these features seem to be small for cortical slices, at least compared to the effects from the saline cover, cf. Figs. 7 and 13H, I.

The point-electrode approximation gives accurate results when the current sources are positioned far away from the electrode contact (Moulin et al. 2008). However, for sources close to the contact, this approximation breaks down due to distortions of the electrical field around the highly conductive contact surface. Most of this effect can be accounted for by simply averaging the point-electrode MoI expression across the electrode surface, i.e., the *disc-electrode* approximation in Eq. 22. With this approach we found that the deviation of the computed potentials from the corresponding FEM results was less than 10 % for source distances larger than half the electrode radius, cf. Fig. 5.

In line with the findings for test current sources discussed above, we found that a saline cover reduces the amplitude of a spike, i.e., the extracellular signature of an action potential, but also makes it more local in the lateral directions. Thus a saline cover will in principle make it easier to estimate the lateral position of the spiking neuron. However, the effect of the saline cover is smaller for a spike (cf. Fig. 11) (where the net transmembrane current averaged across the neuronal membrane is zero (Pettersen and Einevoll 2008)) than for the monopolar test source (cf. Fig. 9). In contrast to the effects from the saline cover, a putative thin saline layer sandwiched between the MEA-chip and the brain slice will not only reduce the spike amplitude, but also blur it, i.e., make it less confined laterally, cf. Fig. 12.

The recorded MEA potential (here denoted *local field potential (LFP)*) simulated in a cortical network model comprising more than 3000 neurons, was seen to be affected by the saline cover, but essentially unaffected by the expected anisotropy and inhomogeneity of the electrical conductivity in a cortical slice (Goto et al. 2010), cf. Fig. 13. The estimated *current-source density (CSD)*, however, is essentially unaffected by the presence of the saline cover. This simplifying feature can be understood from the fact that (i) the CSD is essentially given by the curvature, i.e., double-spatial derivative, of the LFP, and (ii) that the curvature of the LFP contribution from the image current sources reflecting the saline cover will, as shown here, be very small in the MEA-chip plane. Thus in ‘forward-inverse’ CSD estimations using the iCSD (Pettersen et al. 2006; Łęski et al. 2007, 2011) or kCSD (Potworowski et al. 2012) methods on recorded MEA potentials in experiments with a saline cover, it may in practice be sufficient to use a forward model neglecting the saline cover in constructing the CSD estimator (like in the present kCSD _0_ method). Thus the methods developed previously for CSD estimation of *in vivo* LFP recordings, such as 2D iCSD (Łęski et al. 2011) and kCSD (Potworowski et al. 2012) methods, for example, are still applicable to MEA recordings (except for an overall amplitude factor of two due to the effectively insulating MEA chip). It should be noted that these conclusions are expected to be quite general: even if the biological realism of the present cortical network model can be questioned, the generated CSD and LFP data is still expected to be well suited for testing the merit of the CSD analysis method itself (Pettersen et al. 2008; Denker et al. 2012).

In the present example applications, the reference electrode, i.e., ground, has been assumed to be at the outer rim of the simulation grid for FEM and infinitely far away for MoI. In some MEA applications, however, the reference electrode is embedded directly in the glass substrate of the MEA. As both our methods (FEM and MoI) compute the potentials at everywhere on the glass substrate, i.e., MEA plane, the definition of ground can easily be changed from the present choices by instead computing the difference between the MEA potentials at the recording electrode contacts and the reference electrode.

The present work has focused on MEAs with flat electrodes embedded in the chip surface. However, the present approach can also be used to develop similar tools for MEA slice recordings with 3D electrodes, e.g., tip-shaped or nail-like, protruding from the MEA-chip surface, and where the detailed electrical field pattern around the microelectrode contacts will be different (Heuschkel et al. 2002; Hai et al. 2010). Likewise, in the present work we have assumed the voltage-measurement system at the microelectrode to be ‘ideal’, i.e., having infinite input impedance (Moulin et al. 2008). This implies that the only effect from the microelectrode contacts on the surrounding electrical field comes from the metal-like boundary conditions imposed at the microelectrode contact surface. However, the formalism can be modified to situations with non-ideal recording systems so that the electrode-tissue interface impedance is not negligible compared to the overall impedance of the voltage-measurement system, see Moulin et al. (2008).

Another important application of MEAs is the recording of activity from neuronal cultures (Gal et al. 2010; Tetzlaff et al. 2010; Lambacher et al. 2011; Hierlemann et al. 2011). Here the neurons are grown on top of, or around (Nam et al. 2006; Hai et al. 2010), the MEA contacts, and the neuronal morphologies, crucial for computing MEAs potentials, will be modified accordingly (see, e.g., Figs. 10 and 19 in Hierlemann et al. (2011)). However, the basic measurement physics is unchanged, and with additional assumptions about the detailed shapes of the morphologies in this context, the present approach can be used also here.

## Information Sharing Statement

The computer code for using the new methods, i.e., the new Python toolbox ViMEAPy and upcoming new releases of kCSD estimation toolboxes in Matlab and Python, will be made publicly available at the INCF Software repository (software.incf.org) and other relevant repositories.
